# 11 years of tracking aid to reproductive, maternal, newborn, and child health: estimates and analysis for 2003–13 from the Countdown to 2015

**DOI:** 10.1016/S2214-109X(16)30304-7

**Published:** 2016-12-10

**Authors:** Christopher Grollman, Leonardo Arregoces, Melisa Martínez-Álvarez, Catherine Pitt, Anne Mills, Josephine Borghi

**Affiliations:** a Department of Global Health and Development, London School of Hygiene & Tropical Medicine, London, UK

## Abstract

**Background:**

Tracking aid flows helps to hold donors accountable and to compare the allocation of resources in relation to health need. With the use of data reported by donors in 2015, we provided estimates of official development assistance and grants from the Bill & Melinda Gates Foundation (collectively termed ODA+) to reproductive, maternal, newborn, and child health for 2013 and complete trends in reproductive, maternal, newborn, and child health support for the period 2003–13.

**Methods:**

We coded and analysed financial disbursements to reproductive, maternal, newborn, and child health to all recipient countries from all donors reporting to the creditor reporting system database for the year 2013. We also revisited disbursement records for the years 2003–08 and coded disbursements relating to reproductive and sexual health activities resulting in the Countdown dataset for 2003–13. We matched this dataset to the 2015 creditor reporting system dataset and coded any unmatched creditor reporting system records. We analysed trends in ODA+ to reproductive, maternal, newborn, and child health for the period 2003–13, trends in donor contributions, disbursements to recipient countries, and targeting to need.

**Findings:**

Total ODA+ to reproductive, maternal, newborn, and child health reached nearly US$14 billion in 2013, of which 48% supported child health ($6·8 billion), 34% supported reproductive and sexual health ($4·7 billion), and 18% maternal and newborn health ($2·5 billion). ODA+ to reproductive, maternal, newborn, and child health increased by 225% in real terms over the period 2003–13. Child health received the most substantial increase in funding since 2003 (286%), followed by reproductive and sexual health (194%), and maternal and newborn health (164%). In 2013, bilateral donors disbursed 59% of all ODA+ to reproductive, maternal, newborn, and child health, followed by global health initiatives (23%), and multilateral agencies (13%). Targeting of ODA+ to reproductive, maternal, newborn, and child health to countries with the greatest health need seems to have improved over time.

**Interpretation:**

The increase in reproductive, maternal, newborn, and child health funding over the period 2003–13 is encouraging. Further increases in funding will be needed to accelerate maternal mortality reduction while keeping a high level of investment in sexual and reproductive health and in child health.

**Funding:**

Subgrant OPP1058954 from the US Fund for UNICEF under their Countdown to 2015 for Maternal, Newborn and Child Survival Grant from the Bill & Melinda Gates Foundation.

## Introduction

Since 1990, maternal and child mortality rates have fallen by about half, with the largest reductions occurring since 2000.^1^ However, only a third of countries with the greatest mortality burden (the 75 Countdown priority countries) achieved Millennium Development Goal 4 (MDG 4; to reduce the mortality rate in children younger than 5 years by two-thirds between 1990 and 2015), and only 6% achieved MDG 5 (to reduce the maternal mortality ratio by three-quarters).^1^ Part of the mortality reductions achieved has been attributed to increased coverage of life-saving interventions.^2^ Although vaccination coverage and malaria intervention coverage have increased substantially, coverage of family planning and safe motherhood interventions remain inadequate, and wide inequalities in coverage persist.^1^ Availability of adequate financial resources is a factor enabling progress towards improved health outcomes, through funding quality health services.^3^, ^4^, ^5^ In many low-income countries, donor funding represents a substantial share of health sector financing, amounting to an average of 33% of total health expenditure across all low-income countries in 2013.^6^

As part of the Countdown initiative, we have tracked aid flows to maternal, newborn, and child health, reporting findings every 2 years since 2006. From its original focus on tracking aid flows to maternal, newborn, and child health, our resource tracking exercise extended in 2009 to include reproductive and sexual health (reproductive, maternal, newborn, and child health). Since we began tracking resource flows, the Institute for Health Metrics and Evaluation (IHME) has begun tracking development assistance to the health sector^7^ and the Partnership for Maternal, Newborn and Child Health tracks aid to reproductive, maternal, newborn, and child health.^8^ The resource flows project of the United Nations Population Fund (UNFPA) and the Netherlands Interdisciplinary Demographic Institute, tracking international population assistance, has been underway since 2002,^9^ and the Organisation for Economic Co-operation and Development recently began requesting that donors report on a reproductive, maternal, newborn, and child health policy marker as part of their official reporting. Unlike other initiatives, we take a detailed approach to resource tracking, coding record by record with the use of project descriptions, based on a clear conceptual framework and coding scheme. This enables us to maintain a highly disaggregated classification of projects and to estimate the full value of resources benefiting reproductive, maternal, newborn, and child health, including a proportion of aid to the health sector, and to the humanitarian and other sectors.^10^, ^11^, ^12^, ^13^, ^14^, ^15^, ^16^

**Research in context**

**Evidence before this study**

We searched for published literature in Pubmed using the terms: global AND (“external funding” OR “resource tracking” OR “official development assistance”) AND (maternal OR reproductive OR child OR newborn) for the period 2003–2016. We also reviewed Institute of Health Metrics and Evaluation (IHME), Partnership for Maternal, Newborn and Child Health (PMNCH) and the United Nations Population Fund (UNFPA) and the Netherlands Interdisciplinary Demographic Institute (NIDI) reports on aid flows, downloaded from the IHME, WHO and Resource Flows project websites. Several estimates exist of official development assistance plus grants from the Bill & Melinda Gates Foundation (ODA+) to reproductive, maternal, newborn and child health, including the past publications of the Countdown project, two studies examining external funding to reproductive health in conflict-affected settings, and estimates from the Institute for Health Metrics and Evaluation, Partnership for Maternal, Newborn and Child Health and UNFPA/NIDI. Past Countdown publications have not previously included updated estimates of ODA+ from previous years.

**Added value of this study**

This study extends the analysis of ODA+ to reproductive, maternal, newborn and child health to include disbursements made in 2013, disbursements made in 2003–12 but reported later, and corrections to inconsistencies in coding over the whole period of the Countdown project. This has resulted in a complete dataset of ODA+ disbursements for reproductive, maternal, newborn, and child health for the period 2003–13, as reported to the Organisation for Economic Co-operation and Development creditor reporting system, allowing for the first time analyses of trends in complete ODA+ to reproductive, maternal, newborn, and child health for the full period.

**Implications of all the available evidence**

We found that ODA+ for reproductive, maternal, newborn, and child health increased alongside ODA+ to the health sector, and faster than overall increases in ODA+. Increases have been greatest for child health, mainly relating to immunisation, and reproductive and sexual health, primarily relating to HIV; increases for maternal and newborn health have been smaller. Targeting to health need seems to have improved among recipient countries, particularly from 2009 to 2013. Different tracking exercises give a wide range of estimates.

This study presents an overview of trends in funding to reproductive, maternal, newborn, and child health over the period 2003–13. Using data reported by donors in 2015, we provide estimates for 2013 and updated estimates for 2003–12, including complete trends in reproductive and sexual health, which had previously only been reported from 2009. We present global trends, focusing particularly on the 75 Countdown priority recipient countries, where over 95% of all maternal and child deaths occur, and assess trends in targeting to health need.^17^, ^18^ We reflect on findings over the period 2003–13 and at the end of the MDG era, to inform accountability exercises related to the Sustainable Development Goals.

## Methods

### Study design

We downloaded official development assistance (ODA) disbursement data for the period 2003–13 from the creditor reporting system of the Organisation for Economic Co-operation and Development on Jan 7, 2015. We obtained data for disbursements made by the Global Alliance for Vaccines and Immunization (GAVI) for 2003–06 directly from GAVI as they were not available through the creditor reporting system (Mocova D, GAVI**,** personal communication). We tracked disbursements to all recipient countries (147 countries in 2013, plus 17 regional recipients and unspecified bilateral disbursements) from all donors reporting ODA disbursements to the creditor reporting system (64 donors in 2013) and from the Bill & Melinda Gates Foundation, which has reported disbursements to the creditor reporting system from 2009 onwards. Although we considered private grants rather than ODA, we included Bill & Melinda Gates Foundation grants in our analyses and we refer to ODA+ when reporting results that include these grants. Other official flows were excluded.

We classified aid by donor type, using the Organisation for Economic Co-operation and Development definitions to avoid double counting. We classified as bilateral aid disbursements for which the donor country specifies the recipient country or purpose of aid, and as multilateral aid disbursements by multilateral institutions where the multilateral institution specifies the recipient country or purpose of aid. We allocated unspecified regional disbursements to countries within the given region based on their year-specific share of direct regional disbursements to reproductive, maternal, newborn, and child health.^13^ Where the recipient was reported as “bilateral, unspecified”, we included disbursements by allocating shares of such disbursements to each country receiving direct disbursements, based on their year-specific shares of direct disbursements.

### Data coding

Our coding framework classifies activities into 27 Countdown codes according to the degree to which they respectively benefit reproductive and sexual health, maternal and newborn health, and child health. Reproductive and sexual health expenditures are defined as expenditures on family planning, sexual health, and sexually transmitted infections, including HIV.^12^ Maternal and newborn health expenditures cover activities to restore, improve, and maintain the health of women and their newborn babies during pregnancy, childbirth, and the first month of life.^16^ Expenditures for child health include activities to restore, improve, and maintain the health of children up to 5 years of age.^16^ Where age was not specified, “child” was assumed to refer to children aged under 5 years. Funding for research activities was excluded.

We included funds explicitly earmarked for reproductive, maternal, newborn, and child health and funds for other activities considered to benefit reproductive, maternal, newborn, and child health, such as funds for health system strengthening, general health-care provision, general budget support, and basket or sector funding, as well as some health-condition-specific funding—eg, funding for malaria and HIV. Funds to these broader expenditure categories were allocated to reproductive, maternal, newborn, and child health based on a set of rules reflecting the proportion of the disbursement considered to benefit reproductive, maternal, newborn, and child health (appendix p 1). For condition-specific funding, country-specific and region-specific rules were based on the proportion of the population aged younger than 5 years (malaria, other infectious diseases, and conditions affecting general population health); the proportion of people living with HIV who were children aged younger than 5 years and women aged 15 years or older (general HIV support); and the prevalence of women aged 15–49 among people living with any of four sexually transmitted infections (*Chlamydia trachomatis, Neisseria gonorrhoeae, Treponema pallidum*, and *Trichomonas vaginalis*).^12^ For general budget support, the rule was based on the proportion of government spending that goes to health and was obtained from the National Health Accounts database.^6^ The allocation of health systems funds and basket or sector funding to reproductive, maternal, newborn, and child health was the same for all countries, with estimates of spending related to reproductive, maternal, newborn, and child health based on the scientific literature.^16^

We reviewed and coded 231 398 disbursement records for the year 2013 across all sectors according to a previously developed framework.^12^, ^16^ The only addition to the coding method used previously in Countdown analyses was that in this iteration, flags were added to the data to be coded to indicate the presence of key terms related to the reproductive, maternal, newborn, and child health codes to be assigned. These flags were generated through searches for terms in the three descriptive fields—project title, short description, and long description—and used to indicate that a record was likely to be related to a particular Countdown code. This aimed to reduce the cognitive load and increase the speed of manual review, and all records were manually coded regardless of the presence of flags. Where there was any ambiguity in coding, we clarified and revised the coding framework (appendix p 2). To create a fully coded dataset for 2003–13, we combined these records with records coded in previous rounds of the Countdown project, covering 2003–12. As the reproductive and sexual health codes were used only from 2009 onwards, we also revisited disbursement records for the years 2003–08 across all sectors, and re-coded those eligible for a reproductive and sexual health activity code.

Donors not only add disbursement data to the creditor reporting system database but also might change previously reported disbursements for earlier years. Until now, analyses of disbursement trends did not comprehensively update previous analyses to account for these changes, nor were thorough consistency checks undertaken to compare the assignment of codes over time.^10^, ^11^, ^12^, ^13^, ^14^, ^16^ In each of the six rounds of coding, usually one, but up to four researchers, coded the newest 1 or 2 years of data. To ensure coding consistency over time we identified records within the Countdown database that were identical on five fields (creditor reporting system purpose code, project title, short description, long description, and donor), but had been assigned different Countdown codes. We identified 82 150 discrepant records in 6616 unique groups of records that were identical on the five fields. Where one record in a group related to a 2013 disbursement, all records in that group were assigned the 2013 code; where all discrepant records in a group related to disbursements between 2003–12, CG or LA reviewed and reconciled codes.

To update the Countdown database for the period 2003–13, we matched the Countdown database to the creditor reporting system dataset downloaded in January 2015 on the five fields. 137 909 records in the 2015 creditor reporting system had a null or zero disbursement value and were coded zero. Where records in the 2015 creditor reporting system were successfully matched on the five fields, they were assigned the code from the Countdown database; 1 428 027 records were coded in this way. We manually coded the remaining 556 587 unmatched records. We also obtained and coded 1190 records directly from GAVI for the period 2003–06 and added them to the final dataset. We checked the resulting dataset of 2 123 713 coded records for 2003–13 for coding inconsistencies identified during the dataset preparation and corrected codes for 11 136 records. For example, we found that vaccination for yellow fever had in some years been coded as only benefiting children (code 415); such records were assigned the correct code for vaccinations benefitting the general population (436; appendix p 2). The fully coded dataset is available online.

### Statistical analysis

We analysed trends in ODA+ and ODA+ to the health sector over the period 2003–13. ODA+ to the health sector was defined as funding falling under the following purpose codes in the Creditor Reporting System: Health (code 120) and Population Policies, Programmes, and Reproductive Health (code 130). All creditor reporting system purpose codes are listed online. Unlike our estimates of ODA+ to reproductive, maternal, newborn, and child health, the estimate of ODA+ to health does not include ODA+ benefiting health reported to other sectors. We analysed trends in donor funding to reproductive, maternal, newborn, and child health for the period 2003–13. We examined variation in reproductive, maternal, newborn, and child health funding amounts over time by donor and donor type (bilateral, multilateral, global health initiative, and private foundation); by funding modality (pooled funding versus project funding) and project type; and by recipient countries and regions. Data were transferred to Microsoft SQL Server Management Studio for data management. Data were exported to Microsoft Excel for coding. Disbursement data were converted to constant 2013 US dollars using the donor-specific Development Assistance Committee deflators.

We explored whether donors targeted their ODA+ to countries with the greatest health needs and whether targeting changed over time. For each of the years 2003–13, we calculated Spearman's correlation between ODA+ to child health per child and mortality in children younger than 5 years, ODA+ to maternal and newborn health per birth and maternal mortality, ODA+ to reproductive and sexual health per woman of reproductive age and HIV prevalence, and ODA+ to reproductive and sexual health per woman of reproductive age and female life expectancy at birth. We obtained data for mortality in children younger than 5 years, maternal mortality, HIV prevalence, and female life expectancy from the World Bank's World Development Indicators, and population data from the UN World Population Prospects.^19^ Rather than pick an arbitrary lag period, we used disbursements and health-need indicators from the same year.

### Role of the funding source

The funders of the study had no role in the study design, data collection, data analysis, data interpretation, or writing of the report. The corresponding author had full access to all the data in the study and had final responsibility to submit for publication.

## Results

**Figure 1:**
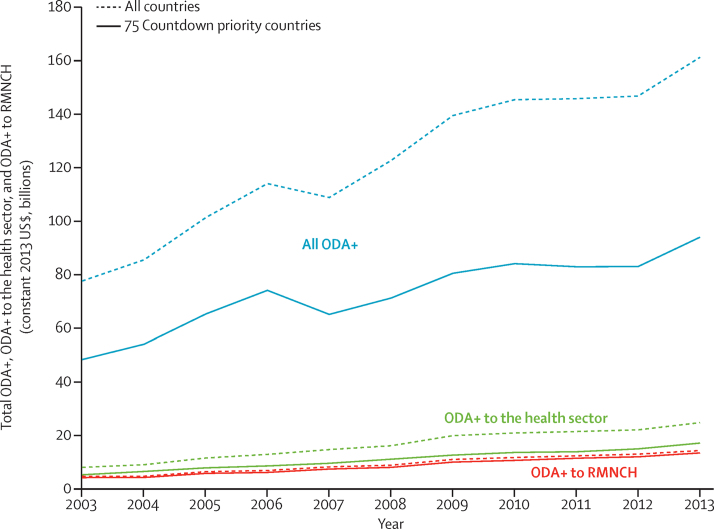
Total ODA+, ODA+ to the health sector, and ODA+ to RMNCH from 2003 to 2013 ODA+ to the health sector includes ODA and private grants coded by the donors as supporting the health sector, population policies or programmes, or reproductive health as defined within the Organisation for Economic Co-operation and Development's creditor reporting system database (purpose codes 120 and 130, respectively). ODA+=official development assistance plus grants from the Bill & Melinda Gates Foundation. RMNCH=reproductive, maternal, newborn, and child health.

Total ODA+ to all sectors was estimated at US$161 billion in 2013, with ODA+ to the health sector at $24 billion (15% of total ODA+), a 10% and 12% increase relative to 2012, respectively (figure 1). Over the period 2003–13, total ODA+ to all sectors more than doubled (increase of 108%) and ODA+ to the health sector increased more than three-fold (219%), with an increase in the share of ODA+ allocated to the health sector over time (from 10% in 2003 to 15% in 2013; appendix p 4).

Total ODA+ to reproductive, maternal, newborn, and child health reached almost US$14·0 billion in 2013, of which 48% supported child health ($6·8 billion), 34% supported reproductive and sexual health ($4·7 billion), and 18% maternal and newborn health (US$2·5 billion; appendix p 5). ODA+ to reproductive, maternal, newborn, and child health in 2013 was 11% higher than that in 2012, with the greatest increase in child health (18%) and the lowest for reproductive and sexual health (6%). Over the period 2003–13, ODA+ to reproductive, maternal, newborn, and child health more than tripled, increasing by 225%.

ODA+ to reproductive, maternal, newborn, and child health and ODA+ to the health sector increased at a similar rate over the period 2003–13. In 2013, 94% of the value of our estimate of ODA+ to reproductive, maternal, newborn, and child health came from funding to the health sector (within the 120 and 130 purpose codes); this proportion increased slightly over time, ranging from 87–89% in 2003–06 and 90–94% in 2007–13 (data not shown). Child health received the most substantial increase in funding over the period 2003–13 (286%), followed by reproductive and sexual health (194%), and maternal and newborn health (164%).

**Figure 2:**
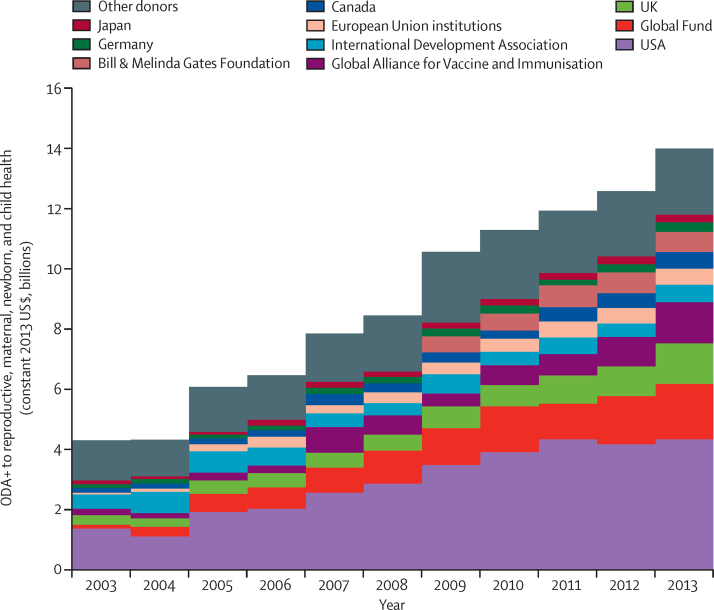
Disbursements of ODA+ to reproductive, maternal, newborn, and child health by donor, for top ten donors and all others from 2003 to 2013 ODA+=official development assistance plus grants from the Bill & Melinda Gates Foundation.

In 2013, bilateral donors disbursed 59% of all ODA+ to reproductive, maternal, newborn, and child health, followed by global health initiatives (23%), and multilaterals (13%; table 1, appendix p 5). The top four donors for reproductive, maternal, newborn, and child health over the period 2003–13 were the USA (US$32 billion), the Global Fund ($11 billion), the UK ($7·3 billion), and GAVI ($6·6 billion; appendix p 8). These were also the four largest donors in 2013 (figure 2, table 1). Bilateral funding grew three-fold, and multilateral funding increased 1·5-fold over the period 2003–13. The UK and Canada increased their ODA to reproductive, maternal, newborn, and child health four-fold over this period, the largest relative increase among bilateral donors. Most Scandinavian countries (Sweden, Denmark, and Finland) decreased their ODA to reproductive, maternal, newborn, and child health, as did the European economies worst affected by the global financial crisis, notably Greece and Spain. The only large increase among multilateral donors was the seven-fold increase from the European Union institutions. Global health initiatives increased funding more than nine-fold, with a 13-fold increase from the Global Fund and a six-fold increase from GAVI (appendix p 6).

**Table 1:** ODA+ to reproductive, maternal, newborn, and child health by donor in 2003, 2008, and 2013 (2013 constant US$, millions), and average annual rate of increase

		**2003**	**2008**	**2013**	**2003–13 average annual rate of change**
Bilateral aid agencies		2855·8	5401·1	8285·0	9%
	Australia	111·3	156·0	217·7	5%
	Austria	4·6	8·0	4·5	0%
	Belgium	46·4	54·2	62·0	2%
	Canada	138·2	286·1	560·4	11%
	Czech Republic	..	..	1·8	..
	Denmark	34·3	52·0	63·0	5%
	Estonia	..	..	0·4	..
	Finland	15·6	30·4	23·7	3%
	France	80·5	64·9	126·1	4%
	Germany	141·4	231·1	324·7	7%
	Greece	29·3	5·6	0·1	−35%
	Iceland	..	..	1·8	..
	Ireland	44·5	73·1	50·3	1%
	Italy	28·7	61·8	39·4	2%
	Japan	117·4	152·2	254·9	6%
	South Korea	0·0	29·2	62·4	..
	Kuwait	..	..	1·3	..
	Luxembourg	0·0	33·8	24·5	..
	Netherlands	145·7	196·8	195·3	2%
	New Zealand	5·7	13·8	13·8	7%
	Norway	68·7	146·1	225·4	10%
	Poland	..	..	0·6	..
	Portugal	3·1	3·1	5·7	5%
	Slovakia	..	..	0·3	..
	Slovenia	..	..	0·4	..
	Spain	56·9	204·7	38·4	−3%
	Sweden	70·9	141·4	173·6	7%
	Switzerland	29·5	36·4	51·1	4%
	United Arab Emirates	..	..	68·0	..
	UK	320·1	543·6	1350·5	12%
	USA	1362·9	2876·8	4342·8	9%
Multilateral aid agencies		1106·2	1329·5	1822·8	4%
	African Development Bank	0·0	0·0	0·2	..
	African Development Fund	15·5	55·5	23·1	3%
	Arab Fund	..	1·1	1·4	..
	Asian Development Bank Special Funds	0·0	0·0	27·0	..
	Arab Bank for Economic Development in Africa	..	..	0·7	..
	European Union institutions	80·7	359·0	532·7	16%
	Global Environment Facility	0·0	0·3	0·4	..
	International Development Association	468·6	412·0	565·6	1%
	Inter-American Development Bank Special Fund	0·0	0·0	35·1	..
	International Monetary Fund (concessional trust funds)	37·2	45·6	33·3	−1%
	OPEC Fund for International Development	..	..	11·7	..
	The Joint United Nations Programme on HIV/AIDS	81·5	61·8	50·1	−4%
	UNDP	..	8·0	7·3	..
	UNFPA	326·8	143·7	150·3	−6%
	The UN Refugee Agency	..	..	0·0	..
	UNICEF	95·9	201·0	165·2	4%
	UN Peacebuilding Fund	..	0·0	0·0	..
	UNRWA	..	41·6	42·9	..
	UN World Food Programme	..	0·0	23·1	..
	WHO	..	..	152·7	..
Global health initiatives		344·8	1719·1	3214·2	19%
	Global Alliance for Vaccines and Immunization	212·1	640·7	1371·4	15%
	Global Fund to Fight AIDS, Tuberculosis and Malaria	132·7	1078·4	1842·8	22%
Private donor		..	..	667·0	..
	Bill & Melinda Gates Foundation	..	..	667·0	..
Total		4306·8	8449·7	13 988·9	9%

Disbursements are in US$ (millions). Average annual rate of change was calculated as the average annual rate of increase necessary starting from the 2003 value to reach the 2013 value; levels of year-on-year change tended to fluctuate. ODA+=official development assistance plus grants from the Bill & Melinda Gates Foundation. OPEC=Organization of the Petroleum Exporting Countries. UNRWA=The United Nations Relief and Works Agency for Palestine Refugees in the Near East.

**Figure 3:**
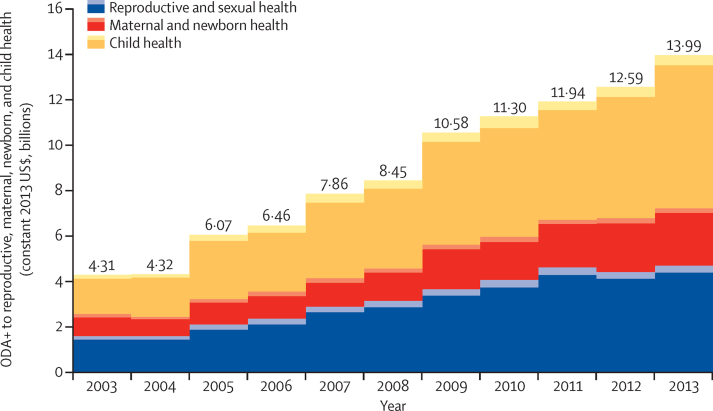
ODA+ to reproductive, maternal, newborn, and child health to priority and non-priority recipients from 2003 to 2013 Darker areas represent 75 Countdown priority recipient countries and lighter areas represent all other non-priority recipient countries. ODA+=official development assistance plus grants from the Bill & Melinda Gates Foundation.

ODA+ to the health sector and to reproductive, maternal, newborn, and child health has been concentrated in the 75 priority countries (figure 3, appendix p 8). Among the Countdown priority countries, ODA+ to reproductive, maternal, newborn, and child health increased between 2012 and 2013 (by 12%), whereas among the non-priority countries it fell for reproductive and sexual health (–6%), maternal and newborn health (–6%), and child health (–2%). A greater share of ODA+ received by the 75 priority countries was disbursed to the health sector compared with non-priority countries (18% *vs* 11%), and ODA+ to the health sector increased more in priority countries over the period 2003–13 than in non-priority countries (243% *vs* 176%). The ratio of reproductive, maternal, newborn, and child health ODA+ to health sector ODA+ was higher in the priority countries than in other countries (78% *vs* 12%). The growth in ODA+ to reproductive and sexual health, maternal and newborn health, and child health over the period 2003–13 was also much greater in priority than non-priority countries: 205% versus 90% for reproductive and sexual health, 305% versus 128% for child health, and 190% versus 31% for maternal and newborn health.

Similar to previous years, in 2013, of the 75 priority countries, Nigeria, Ethiopia, and Kenya received the largest total disbursements of ODA+ to reproductive, maternal, newborn, and child health, while Equatorial Guinea, Turkmenistan, and Gabon received the smallest (table 2; appendix p 8). Over the period 2003–13, Equatorial Guinea and Brazil experienced the largest reductions in ODA+ to reproductive, maternal, newborn, and child health (–80% and −74%, respectively), whereas Somalia and Nigeria received the greatest relative increases, of 878% and 768%, respectively. The total disbursements to reproductive, maternal, newborn, and child health to several recipient countries varied substantially from year to year. For example, disbursements to Botswana more than tripled between 2007 and 2008 from $38 million to $125 million, then fell by more than half between 2009 and 2010, from $140 million to $60 million.

**Table 2:** ODA+ to reproductive, maternal, newborn, and child health to 75 Countdown priority recipient countries in 2003, 2008, and 2013, and average annual increase

	**2003**	**2008**	**2013**	**2003–13 average annual rate of change**
Afghanistan	50·1	258·5	377·0	17%
Angola	40·8	103·7	98·7	7%
Azerbaijan	3·4	7·9	13·9	11%
Bangladesh	179·8	185·8	412·4	7%
Benin	28·6	58·8	80·2	8%
Bolivia	57·4	52·0	45·3	−2%
Botswana	17·4	124·8	51·6	9%
Brazil	19·9	10·5	5·1	−10%
Burkina Faso	36·6	100·6	136·4	11%
Burundi	21·8	58·5	93·5	12%
Cambodia	69·7	92·0	132·6	5%
Cameroon	26·7	42·7	97·8	11%
Central African Republic	8·7	28·6	37·7	12%
Chad	24·2	40·0	105·6	12%
China	83·9	133·9	41·0	−5%
Comoros	4·6	1·7	9·0	5%
Republic of the Congo	6·5	17·5	18·5	8%
Cote d'Ivoire	41·6	80·3	133·8	9%
Korea	3·9	8·7	24·5	15%
Democratic Republic of the Congo	77·3	286·9	549·6	16%
Djibouti	3·0	12·8	9·9	10%
Egypt	47·8	83·2	14·8	−9%
Equatorial Guinea	2·7	11·4	0·5	−12%
Eritrea	33·6	30·7	33·1	0%
Ethiopia	171·2	449·8	872·3	13%
Gabon	2·7	5·0	5·1	5%
Gambia	11·2	11·0	28·2	7%
Ghana	79·8	132·8	257·7	9%
Guatemala	27·1	58·7	41·0	3%
Guinea	21·1	32·6	41·5	5%
Guinea-Bissau	4·7	11·1	27·2	14%
Haiti	45·1	127·2	197·2	12%
India	373·4	509·2	495·4	2%
Indonesia	113·8	136·9	177·3	3%
Iraq	84·0	40·7	28·5	−8%
Kenya	162·7	296·4	755·8	13%
Kyrgyz Republic	28·7	24·1	18·8	−3%
Laos	17·5	27·0	45·8	8%
Lesotho	10·1	25·8	84·2	18%
Liberia	11·3	80·0	94·5	18%
Madagascar	57·3	90·2	143·1	7%
Malawi	105·5	194·9	333·3	9%
Mali	37·7	98·9	199·6	14%
Mauritania	12·3	17·6	17·5	3%
Mexico	10·2	3·7	5·5	−5%
Morocco	29·0	21·3	48·7	4%
Mozambique	136·3	283·1	433·7	9%
Myanmar	24·3	53·4	153·5	15%
Nepal	51·4	76·4	87·1	4%
Niger	23·2	88·4	107·7	13%
Nigeria	139·6	478·2	1211·5	18%
Pakistan	111·8	215·7	554·8	13%
Papua New Guinea	62·1	84·0	126·8	6%
Peru	24·0	37·2	46·6	5%
Philippines	61·9	57·5	82·0	2%
Rwanda	46·5	169·5	217·0	13%
Sao Tome and Principe	2·3	5·5	8·7	11%
Senegal	67·4	82·8	121·4	5%
Sierra Leone	16·6	46·0	95·0	14%
Solomon Islands	7·8	11·4	16·8	6%
Somalia	9·8	43·4	95·7	19%
South Africa	82·4	291·2	478·0	14%
South Sudan	..	..	206·6	..
Sudan	19·3	168·2	160·9	18%
Swaziland	9·6	16·5	52·1	14%
Tajikistan	7·4	20·5	29·7	11%
Tanzania	129·9	413·4	750·1	14%
Togo	10·8	26·5	46·3	12%
Turkmenistan	2·4	2·2	1·9	−2%
Uganda	153·2	234·5	455·2	9%
Uzbekistan	17·9	20·2	24·6	2%
Vietnam	84·1	99·8	151·9	5%
Yemen	26·7	53·5	202·4	17%
Zambia	118·6	242·0	422·4	10%
Zimbabwe	53·0	87·2	274·8	13%
Total	3805·9	7634·6	13 056·6	10%

Disbursements are in US$ (millions). Average annual rate of change was calculated as the average annual rate of increase necessary starting from the 2003 value to reach the 2013 value; in fact, levels of year-on-year change tended to fluctuate. ODA+=official development assistance plus grants from the Bill & Melinda Gates Foundation.

In 2013, ODA+ to child health per child younger than 5 years and to maternal and newborn health per livebirth among the 75 priority countries was highest in São Tomé and Príncipe ($130·05 per child and $350·58 per livebirth) and lowest in Mexico ($0·07 per child and $0·24 per livebirth). The largest change in ODA+ to child health per child over the period 2003–13 was seen in Lesotho which increased from $6·86 to $103·39 per child younger than 5 years (appendix p 11). The largest change in ODA+ to maternal and newborn health per livebirth was in São Tomé and Príncipe, which increased from $112·97 to $350·58 (appendix p 14). In 2013, ODA to reproductive and sexual health per woman aged 15–49 was highest in Swaziland ($107·14) and lowest in Brazil ($0·02). The largest change in ODA+ to reproductive and sexual health per woman aged 15–49 years over the period 2003–13 was seen in Swaziland where it increased from $20·14 to $107·14 (appendix p 17).

Funding to child health and maternal and newborn health has become increasingly targeted to recipient countries with higher mortality (table 3). Similarly, funding to reproductive and sexual health appears increasingly correlated with higher HIV prevalence over time, whereas there is little change in the correlation between reproductive and sexual health funding and female life expectancy. At the end of the period, many countries with lower need were still receiving substantially more funding per person than other countries with higher need (appendix p 21).

**Table 3:** Spearman correlation coefficients of per-person disbursements with measures of health need

	**Health need**	**2003**	**2004**	**2005**	**2006**	**2007**	**2008**	**2009**	**2010**	**2011**	**2012**	**2013**
ODA+ to child health per child younger than 5 years	Mortality rate in children younger than 5 years	0·3107	0·3799	0·2785	0·2748	0·2911	0·3236	0·3107	0·3599	0·3523	0·3471	0·4529
ODA+ to maternal and newborn health per livebirth	Maternal mortality ratio	0·1709	0·2889	0·2066	0·1554	0·2186	0·2049	0·2179	0·2382	0·2676	0·2867	0·3678
ODA+ to reproductive and sexual health per woman aged 15–49 years	HIV prevalence	0·4811	0·6199	0·5149	0·5539	0·5163	0·5008	0·5462	0·5889	0·577	0·5369	0·6113
ODA+ to reproductive and sexual health per woman aged 15–49 years	Female life expectancy	−0·591	−0·6272	−0·5959	−0·6261	−0·58	−0·6133	−0·6413	−0·6279	−0·5969	−0·584	−0·6092

ODA+=official development assistance plus grants from the Bill & Melinda Gates Foundation.

**Figure 4:**
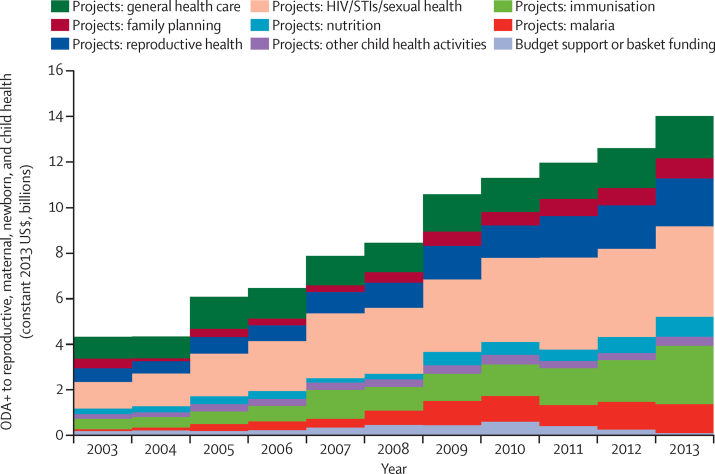
Disbursements of ODA+ to reproductive, maternal, newborn, and child health from all donors to 75 priority recipient countries, by area of spending, 2003–13 ODA+=official development assistance plus grants from the Bill & Melinda Gates Foundation. STI=sexually transmitted infection.

As in previous years, almost all (99%) ODA+ to reproductive, maternal, newborn, and child health in 2013 was channelled as projects, rather than general or sectoral budget support or through pooled funding mechanisms (figure 4, appendix p 20). Funds to HIV continued to comprise the largest share of project funding to reproductive, maternal, newborn, and child health (28%), with almost all of these funds being general-population disbursements not earmarked to reproductive, maternal, newborn, and child health. Funding to immunisation constituted the next largest area of project funding (18%), followed by reproductive health funds (assigned equally between maternal and newborn health and reproductive and sexual health; 15%), and funding for general health-care systems (13%). The largest relative increase in disbursements over the period 2003–13 was for malaria and HIV funding, increasing 14-fold and eight-fold, respectively. Disbursements for immunisation activities also increased by 471% (figure 4, appendix p 20). Over the period 2003–2013, HIV received the largest amount of funds ($2·9 billion), followed by health systems ($1·6 billion), and reproductive health and immunisation (both at $1·3 billion). Although a low share of overall funds, ODA+ to both nutrition and family planning has continued to grow over the past few years, reaching just under $900 million in 2013 in each case.

## Discussion

This study extends previous Countdown tracking work by examining resource flows to reproductive and sexual health before 2009, providing an 11 year time series for reproductive, maternal, newborn, and child health, and incorporating changes to donors' previously reported data. Over the period 2003–13, donors increasingly prioritised the health sector within overall ODA+. ODA+ to reproductive, maternal, newborn, and child health increased at a similar rate to ODA+ to the health sector, with both more than tripling over the 11 year period. Over the period of analysis, children aged between 1 month and 5 years benefited from half of the funding to reproductive, maternal, newborn, and child health and from the largest increase in donor funding, which was driven by substantial increases in funding for malaria and immunisation projects. The trends in funding are also a function of the evolving landscape of donors globally, with the sizable growth in funding from global initiatives such as the Global Fund and GAVI in financing malaria and HIV projects benefiting reproductive, maternal, newborn, and child health. While funding to maternal and newborn health increased substantially over the period, this group benefited from smaller increases than did child health and reproductive and sexual health. There were sustained increases in health system strengthening, which is essential to overcome the bottlenecks in reproductive, maternal, newborn, and child health services.^20^ The MDG final report reported greater progress towards MDG 4 on child mortality than on MDG 5 on maternal mortality,^21^ while post-neonatal child mortality has declined substantially faster than neonatal mortality in the poorest countries.^22^ Further funding increases are needed to accelerate maternal and neonatal mortality reduction, while maintaining a high level of investment in child health.^23^

Donors have been prioritising their aid flows to overall health and to reproductive, maternal, newborn, and child health towards priority countries, which had a much greater growth in related ODA+ over the past 11 years than non-priority countries. However, low and decreasing budget support and the high level of donor-driven project funding limit the potential for the strategic allocation of funds to national priorities. There is substantial volatility in year-on-year total disbursements to recipient countries, which might reflect unpredictability in funding flows, or could result from funds for large multiyear projects being disbursed in a single year.

The apparent improvement in targeting of ODA+ to reproductive, maternal, newborn, and child health to countries with the greatest health needs is consistent with previous findings on targeting of ODA to HIV.^10^, ^13^, ^24^ These trends are encouraging, but should be interpreted cautiously. In previous studies, we considered whether aid was targeted, reporting improvements in targeting at three timepoints. This was done by means of a series of linear regressions considering the size of the coefficient together with that of the R^2^ as an indicator of the degree of targeting, where larger values were interpreted as corresponding to greater targeting.^10^, ^11^ However, with an 11 year time series, such analyses would be inappropriate as the error terms in a series of univariate regressions would be auto-correlated, leading to a biased coefficient. A systematic assessment of the association between aid and recipient-country need and other characteristics, with the use of more complex econometric models to adjust for auto-correlation, would be an important area for future research. Moreover, improved targeting at the global level guarantees neither appropriate funding to priority needs at the national level, nor appropriate subnational distribution of funds.

Our consistency checks, updating of the underlying data, and recoding to include estimates for reproductive and sexual health before 2009, have led us to revise reported estimates for the years 2003–12. For example, our estimates for 2011 and 2012 are lower than previously reported^10^ for ODA+ to reproductive and sexual health and to child health mainly because of corrections we made to coding after the consistency check of historical data which showed that in the past some records relating to food and humanitarian aid were coded as basic health care; these ought to have been excluded according to our coding scheme. Changes to donor reporting of project descriptions led to some changes. For some donors, our estimates exceeded those previously reported, largely because donors retrospectively added projects to the database. For example, we observed an eight-fold increase in the number of projects reported for maternal, newborn, and child health for the years 2004 and 2005 by Norway in 2015 compared with when these years were initially coded, and a 260% increase in the disbursement value to maternal, newborn, and child health. For some donors, all or nearly all of the disbursements for years 2003–12 were added retrospectively.

Our estimates of donor assistance to maternal, newborn, and child health are $0·4 billion lower than the IHME estimates for the year 2013 of $9·7 billion (in 2013 prices). However, IHME estimated a much lower increase in maternal, newborn, and child health funding over the period 2003–13, 93% compared with our estimate of 242%, which we attribute to important underlying differences in the concepts measured. The Partnership for Maternal, Newborn and Child Health estimated total official development assistance to reproductive, maternal, newborn, and child health among the 75 priority countries at $12·1 billion (in 2013 prices), $1 billion less than estimated here, an increase of 116% between 2006 and 2013, compared with our estimate of 127% over that period. UNFPA and the Netherlands Interdisciplinary Demographic Institute estimated international population assistance at $11·0 billion in 2012, a 163% increase relative to 2003.^9^ Their definition of population activities includes the components of reproductive, maternal, and newborn health we analysed and valued at $6·8 billion for 2012, with a 166% increase over the same period, and support for demographic and programme-related data collection and analysis, research, policy development, and training and reporting; they exclude child health activities. Data for 2013 are not yet available. Patel and colleagues^25^ tracked ODA to reproductive health between 2002 and 2011 and found a 298% increase in 18 conflict-affected countries, which they contrasted with a 323% increase in 36 non-conflict-affected least developed countries over the same period. In December, 2014, the Organisation for Economic Co-operation and Development introduced a policy marker for reproductive, maternal, newborn, and child health, which donors can voluntarily use to mark their disbursements as contributing to reproductive, maternal, newborn, and child health. As of March 2016, only half of donors had reported on this policy marker for 2013, and so no time trends were available.

The various approaches to resource tracking differ substantially in their coding methods—Countdown used manual review, IHME used automated key-term searches, and the Partnership for Maternal, Newborn and Child Health used the purpose codes reported by donors to the creditor reporting system. Given these differences, the similarity of the Countdown and IHME estimates for maternal, newborn, and child health might be a coincidence. We report elsewhere a detailed comparison of the methods used by the Countdown, IHME, and the Partnership for Maternal, Newborn and Child Health initiatives, and the creditor reporting system purpose codes and reproductive, maternal, newborn, and child health policy marker, commenting on their respective strengths and making recommendations for future tracking exercises (unpublished).

Our analysis has several limitations. Our method of manual coding relies on human interpretation of a coding scheme, which makes it difficult to replicate because it is subjective and highly labour-intensive. It is also dependent on the way donors describe projects, and on changes made to such descriptions over time. In some cases, projects that were previously described as being relevant to reproductive, maternal, newborn, and child health by donors, and included in our Countdown estimates, were subsequently modified within the creditor reporting system database by donors, such that they no longer were eligible for a Countdown code. Additionally, different people undertook the coding over time, which might have introduced bias. We hope to have minimised this through our standard coding framework and the consistency checks done in the data preparation. Our approach to allocating disbursements where the recipient was “bilateral, unspecified” or was a region might not give entirely accurate results for each recipient, but to exclude these would have led to a large underestimate of ODA+ to reproductive, maternal, newborn, and child health. It is very unlikely that such disbursements were distributed by recipient such as to change the interpretation. More broadly, external funding to reproductive, maternal, newborn, and child health is only one source of country-level funds. Maintenance of gains already made, and further improvements in health outcomes, also depend on domestic funding and out-of-pocket expenditures, including remitted money,^26^ which are not included in this study but are important for funding health systems across the world. Country case studies have reported on domestic financing levels to reproductive, maternal, newborn, and child health in some of the Countdown priority countries.^23^, ^27^, ^28^ In addition to reporting on trends in ODA+, it is also important to consider how disbursements reflect aid effectiveness principles. Recent initiatives, including Every Woman Every Child, refer to reproductive, maternal, newborn, child, and adolescent health. While aspects of adolescent reproductive and sexual health are included in our coding framework, future resource-tracking work could include a wider range of adolescent health activities.

The sustained increase in reproductive, maternal, newborn, and child health funding over the period 2003–13, and possible improved targeting to need, is encouraging. However, substantial unexplained variation between countries remains and further research is needed to assess whether these increases are effective in improving health outcomes.^29^ Donor reporting to the creditor reporting system has improved over time, perhaps in response to tracking exercises, which is encouraging, but it is important that further efforts are made to ensure donors report accurate, complete, consistent, and timely data. Changes to the way donors describe and label projects affects whether they are coded as benefiting reproductive, maternal, newborn, and child health. Major discrepancies between different published estimates is a cause of considerable concern. Systematic comparison of the available tracking methods will help to identify an optimum approach in terms of timeliness and accuracy going forward, and this has been investigated in another study (unpublished).
