# Supplementary material for: 11 years of tracking aid to reproductive, maternal, newborn, and child health: estimates and analysis for 2003–13 from the Countdown to 2015
Source: Lancet Glob Health. 2016 Dec 10;5(1):e104–14. doi: 10.1016/S2214-109X(16)30304-7 (PMC5565636; doi:10.1016/S2214-109X(16)30304-7)
Supplement: Supplementary appendix [file mmc1.pdf]

THE LANCET Global Health

Supplementary appendix

This appendix formed part of the original submission and has been peer reviewed. We post it as supplied by the authors.

Supplement to: Grollman C, Arregoces L, Martínez-Álvarez M, et al. 11 years of tracking aid to reproductive, maternal, newborn, and child health: estimates and analysis for 2003–13 from the Countdown to 2015. *Lancet Glob Health* 2017; **5:** e104–14.

Web Appendix to *11 years of tracking aid to reproductive, maternal, newborn and child health*

Appendix Table 1: Countdown RMNCH codes and respective fractions disbursed to child health (CH), maternal and newborn health (MNH) and reproductive and sexual health (R*)

RMNCH code	Nature of the project	CH fraction	MNH fraction	R* fraction
0	Not relevant to RMNCH			
100	General budget support	Country value	Country value	Country value
200	Health sector budget support	20%	12%	
300	Health basket-funding	40%	8%	
411	Child malaria	100%		
412	HIV/AIDS – prevention of mother-to-child transmission	100%		
413	Nutrition in children	100%		
414	Integrated management of childhood illnesses	100%		
415	Immunisation – excluding polio	100%		
416	Immunisation – polio	100%		
417	Non-specified infectious diseases in children	100%		
418	Non-specified child health	100%		
419	Childhood HIV/AIDS	100%		
421	Reproductive, maternal and neonatal health		100%	
422	Family planning			100%
423	Sexual health			100%
424	Sexual/reproductive health – mixed		50%	50%
431	Maternal and child health	50%	50%	
432	Malaria – generic	Region value	15%	
433	Maternal and child malaria	55%	10%	
434	HIV/AIDS – generic	Country value		Country value
435	Nutrition – generic	50%	50%	
436	Non-specified infectious diseases	Country value		
437	Sexually transmitted infections – generic			Region value
440	General health system – primary health care	40%	8.4%	
450	General health system – hospital level care	11·3%	13·3%	
460	General health system – policy/all levels	19·8%	11·8%	

TRIAL MODE - a valid license will remove this message. See the keywords property of this PDF for more information.

TRIAL MODE - a valid license will remove this message. See the keywords property of this PDF for more information.

Appendix Table 2: Countdown RMNCH codes and definition

RMNCH code	Nature of the project	Description
0	Not relevant to RMNCH	Not related to RMNCH according to our definition. This typically includes activities related to: research, dentistry, tobacco and alcohol/drugs, and non-health-related activities. Research activities are also coded 0, as are conferences unless clearly oriented toward policy or implementation rather than science (e.g. Women Deliver). Core contributions from donors to multilateral agencies such as UNAIDS are coded 0. Everything reported as debt relief (purpose code 600XX) or donor-country promotional activities (purpose code 99820) receive a 0 code.
100	General budget support	Funds disbursed to the central bank or regional governments of the recipient country with no earmarking. Funds should be associated with poverty reduction as opposed to balance of payment support. Includes poverty reduction support credits (PRSCs) but not structural adjustment loans or macroeconomic stabilisation grants. Includes any generic/unspecified “budget support” records.
200	Health sector budget support	Funds disbursed to the central bank or regional governments of the recipient country but earmarked specifically for the health sector. Funds are not earmarked to areas within the health sector. Includes “block grants” to the health sector.
300	Health basket-funding	Funds earmarked to specific areas within the health sector e.g. primary health care. Also includes basic package of health services.
411	Child malaria	Entire project is oriented to malaria activities targeting children from one week up to five years.
412	Prevention of mother-to-child HIV transmission	Entire project is oriented to the prevention of mother-to-child transmission of HIV
413	Childhood nutrition	Entire project is oriented to nutrition activities targeting children under five. Includes breastfeeding and supplementary feeding. Excludes food security, food aid, and school feeding. Unspecified "nutrition" projects are coded as 435 (Nutrition – generic).
414	Integrated management of childhood illnesses	Entire project is oriented to IMCI. Includes integrated community case management (ICCM), integrated management of newborn and child illness (IMNCI), community integrated management of childhood illnesses (CIMCI), "EPI [expanded programme of immunisation] +malaria", young child survival and development (YCSD).
415	Immunisation – excluding polio	Entire project is oriented to immunisation activities and does not specify that the project will only include polio immunization. Includes generic/unspecified vaccine/immunisation projects. Includes TB unless specified for adults. Excludes vaccines in emergency situations (e.g. cholera, yellow fever) or other vaccines that benefit the general population (436). Excludes HPV vaccination (423). Includes "cold chain" etc in 120 codes unless specified for blood (450).
416	Immunisation – polio	Entire project is oriented to polio immunisation activities.
417	Non-specified infectious diseases in children	Entire project is oriented to infectious disease activities other than malaria or HIV/AIDS targeting children under five. The activities target either a single infectious disease or multiple infectious diseases.
418	Non-specified child health	Entire project is oriented to health activities targeting children under five. The activities do not fall within any of the previous child health categories or are not specified. Includes activities to support children’s hospitals and paediatric wards; unspecified child protection listed in 120/130; child survival; and unspecified “child health” projects and health projects for “children”.
419	Childhood HIV/AIDS	Entire project is oriented to treatment of paediatric HIV/AIDS.
421	Reproductive, maternal and neonatal health	Entire project is oriented to maternal and/or neonatal health activities including safe motherhood, care during childbirth and newborn care. Includes training of midwives and obstetricians, congenital syphilis and fistula. Excludes family planning 422, sexual health and sexual and gender-based violence 423, STI-related activities 437, and all projects referring to “reproductive commodities” (e.g. not contraceptives) unless further information is provided to indicate that the commodities fall within our definition of MNH, such as clean delivery kits and other commodities for safe motherhood (else 424). Excludes projects which also mention children, which should be coded as 431. Includes unspecified 13020 projects, but vague "reproductive health" projects are coded as 424 (sexual/reproductive health – mixed).
422	Family planning	Project is oriented to family planning including the provision of and counselling in contraceptive commodities, abortion services, infertility drugs and procedures, and information, education and communication (IEC) activities that support or promote family planning.
423	Sexual health	Entire project is focused on sexual health. Includes behaviour change programmes for safer sexual behaviour, cancers of the reproductive system (including breast, cervical, ovarian, uterine) and HPV vaccination, and sexual and gender-based violence (e.g. rape, incest, sexual trafficking and sexual exploitation) if listed under health or reproductive health purpose codes (120/130) or contains a clear description as addressing a health and not a human rights or other social service aspect if included in the government and civil society codes (150) or other social services codes (160). Includes projects related to female genital mutilation listed in 120/130.

TRIAL MODE - a valid license will remove this message. See the keywords property of this PDF for more information.

424	Sexual/reproductive health – mixed	Includes adolescent/youth sexual and reproductive health programmes or any other programmes that specify SRHR (sexual and reproductive health and rights). Generally includes projects with CRS purpose code 13030 implemented by International Planned Parenthood Federation (IPPF) and many from UNFPA. Use for ‘mixed’ interventions that straddle across code 421 and other RMNH codes – but not code 431. Includes vague "reproductive health" projects and unspecified projects with purpose code 13081; includes vague projects with purpose code 13040 where donor is UNICEF.
431	Maternal and child health	Entire project is oriented to maternal and child health (children older than one week). Includes neonatal only if child is also included. Excludes specific malaria 433 and nutrition 435 projects.
432	Malaria – generic	Entire project is oriented towards malaria without specifying the target population.
433	Maternal/child malaria	Entire project is focused on malaria prevention or treatment activities for women during pregnancy, childbirth and the postnatal period (1 week) for newborns, and projects for both mothers and children under five. Includes all ITN projects, which are assumed to be for mothers and children only. Programmes for children under five only are coded 411.
434	HIV/AIDS – generic	Entire project is oriented towards HIV/AIDS without specifying the target population. TB integrated with HIV is included. Includes "abstinence" etc if described as for HIV prevention. Include generic "high-risk populations" or similar; include sex workers, using an allocation factor if named alongside other high-risk groups; exclude if specifically for men having sex with men or injecting drug users. To the extent possible, exclude projects targeting the socio-economic causes and consequences of HIV, such as livelihoods, education, and orphans and vulnerable children (OVC).
435	Nutrition	Entire project is focused on nutrition activities for women and children. If not explicitly mentioned, both general "nutrition" projects and targeted food supplementation to malnourished persons are assumed to target women and children only. Excludes food security, food aid and school feeding, and excludes projects that mention these alongside "nutrition". Agriculture projects in purpose code 311 is that they are excluded.
436	Non-specified infectious diseases	Project is focused on health activities for which children are likely to be a beneficiary with the same probability as the rest of the population wherein the project does not specify the target group. Includes combined projects targeting multiple infectious diseases (e.g. projects targeting Malaria, TB and HIV jointly). Also includes general population vaccines including emergencies (e.g. cholera), avian flu, land mine clearing and awareness but not solely advocacy or economic support to victims, arsenic poisoning prevention. Includes WASH projects in 140 that mention a targeted disease (e.g. cholera, diarrhoea). Includes eye health projects (in 120) except those addressing non-child problems such as cataracts (0). Excludes diabetes, alcoholism, kidney disease and non-communicable diseases generally unless likely to affect children under five.
437	Sexually transmitted infections – generic	Entire project is focused on sexually transmitted infections (STIs) without specifying the condition or the target population. Includes testing, prevention, treatment and care of STIs, reproductive tract infections and other gynaecological morbidities. Includes HIV alongside STIs, but not HIV alone.
440	General health system – primary health care	Project supports the delivery of integrated services at the primary or basic level of the health system. Such projects do not target a specific disease or area of health. Includes training, infrastructure or resourcing (e.g. drugs) explicitly for primary health care. Includes water, sanitation and hygiene (WASH) activities whose primary stated purpose is to improve human health – not WASH infrastructure alone – as shown by inclusion in the health purpose codes (120); if included in the water purpose codes (140XX) or under the humanitarian purpose codes (720XX), a WASH project would get 440/450/460 if providing WASH for health facilities, 436 if addressing a named disease, and otherwise 0. WASH for schools is excluded; a project for "schools and communities" would get 440 with AF 0.5. Includes humanitarian activities defined vaguely as "health" or implemented by a humanitarian organization whose primary area of activity is health and community health. Includes "health education" projects targeting the population.
450	General health system – hospital level care	Project supports the delivery of integrated services at the hospital or secondary level of the health system. Such projects do not target a specific disease or area of health. Includes blood transfusion services. Includes training, infrastructure or resourcing (e.g. drugs) explicitly for hospital-level care.
460	General health system – policy/all levels	Project supports health system development rather than a specific disease, or demographic group. This could be either at the administrative level (i.e. policy, planning and monitoring) or at the level of service delivery (i.e. health providers). Such projects typically provide financial support for health infrastructure, human resources (which includes salaries as well as training of nurses and doctors including higher education for medical school), drug provision and management, medical equipment, policy development and monitoring systems (e.g. information systems), except where these are more specifically described as coming under 440/450. Includes “health sector reform” projects, core support to international health service delivery NGOs, advocacy and health promotion. Excludes funding for policy in more specific codes, which should take the more specific code. Includes all health insurance projects. Includes "health education" where the object is providers of such education.
999	Disbursement blank/zero	Disbursement is zero or not reported.

TRIAL MODE - a valid license will remove this message. See the keywords property of this PDF for more information.

Appendix Table 3: ODA+ to reproductive, maternal, newborn and child health, total, 75 Countdown priority recipients and non-priority recipients, 2013 US$m, 2003–13

Recipient countries	2003		2004		2005		2006		2007		2008		2009		2010		2011		2012		2013	
**All ODA+ (excluding debt forgiveness)**																						
All	77269		85275		101036		113797		108571		122435		139208		145154		145532		146515		160980	
75 priority	47998		53746		64947		73824		64845		70880		80197		83792		82666		82751		93738	
Non-priority	29272		31529		36088		39972		43726		51555		59011		61362		62866		63764		67242	
**ODA+ to the health sector (% of all ODA+)**																						
All	7646	10%	8672	10%	11119	11%	12506	11%	14291	13%	15742	13%	19502	14%	20506	14%	21046	14%	21715	15%	24387	15%
75 priority	4874	10%	6099	11%	7444	11%	8209	11%	9213	14%	10722	15%	12210	15%	13243	16%	13473	16%	14612	18%	16723	18%
Non-priority	2772	9%	2573	8%	3675	10%	4297	11%	5078	12%	5019	10%	7292	12%	7263	12%	7574	12%	7103	11%	7664	11%
**ODA+ to RMNCH (ratio to ODA+ to the health sector)***																						
All	4307	56%	4322	50%	6069	55%	6457	52%	7862	55%	8450	54%	10577	54%	11299	55%	11940	57%	12588	58%	13989	57%
75 priority	3806	78%	3905	64%	5430	73%	5731	70%	7044	76%	7635	71%	9643	79%	10214	77%	11032	82%	11617	80%	13057	78%
Non-priority	501	18%	417	16%	638	17%	726	17%	818	16%	815	16%	934	13%	1085	15%	908	12%	971	14%	932	12%
**ODA+ to R* (% of ODA+ to RMNCH)**																						
All	1599	37%	1592	37%	2096	35%	2357	37%	2886	37%	3156	37%	3671	35%	4061	36%	4637	39%	4417	35%	4702	34%
75 priority	1445	38%	1445	37%	1872	34%	2117	37%	2637	37%	2877	38%	3371	35%	3730	37%	4311	39%	4109	35%	4411	34%
Non-priority	154	31%	147	35%	223	35%	241	33%	250	31%	279	34%	300	32%	331	31%	326	36%	309	32%	292	31%
**ODA+ to MNH (% of ODA+ to RMNCH)**																						
All	957	22%	849	20%	1133	19%	1200	19%	1248	16%	1424	17%	1947	18%	1910	17%	2094	18%	2374	19%	2530	18%
75 priority	801	21%	742	19%	988	18%	1000	17%	1045	15%	1244	16%	1738	18%	1690	17%	1896	17%	2156	19%	2325	18%
Non-priority	156	31%	107	26%	145	23%	200	28%	203	25%	179	22%	209	22%	221	20%	198	22%	219	23%	205	22%
**ODA+ to CH (% of ODA+ to RMNCH)**																						
All	1751	41%	1881	44%	2839	47%	2900	45%	3728	47%	3870	46%	4959	47%	5328	47%	5209	44%	5797	46%	6757	48%
75 priority	1560	41%	1717	44%	2569	47%	2615	46%	3362	48%	3513	46%	4534	47%	4794	47%	4826	44%	5353	46%	6321	48%
Non-priority	191	38%	163	39%	270	42%	285	39%	366	45%	356	44%	424	45%	533	49%	383	42%	443	46%	436	47%

* 120–130 sector codes comprised 91.1% of the value of all RMNCH disbursements across all years.

Appendix Table 4: Change in ODA+ to reproductive, maternal, newborn and child health, 2003–13

	Change 2003–13	Change 2012–13	Mean annual change 2003–13
**All ODA+ (excluding debt forgiveness)**			
All recipient countries	108%	10%	8%
75 Countdown priority recipients	95%	13%	7%
Non-priority recipients	130%	5%	9%
**ODA+ for health**			
All recipient countries	219%	12%	13%
75 Countdown priority recipients	243%	14%	13%
Non-priority recipients	176%	8%	12%
**ODA+ for RMNCH**			
All recipient countries	225%	11%	13%
75 Countdown priority recipients	243%	12%	14%
Non-priority recipients	86%	-4%	8%
**ODA+ for R***			
All recipient countries	194%	6%	12%
75 Countdown priority recipients	205%	7%	12%
Non-priority recipients	90%	-6%	8%
**ODA+ for MNH**			
All recipient countries	164%	7%	11%
75 Countdown priority recipients	190%	8%	12%
Non-priority recipients	31%	-6%	5%
**ODA+ for CH**			
All recipient countries	286%	17%	15%
75 Countdown priority recipients	305%	18%	16%
Non-priority recipients	128%	-2%	11%

TRIAL MODE - a valid license will remove this message. See the keywords property of this PDF for more information.

Appendix Table 5: Disbursements of ODA+ to RMNCH by donor, 2003–13, $m 2013

	2003	2004	2005	2006	2007	2008	2009	2010	2011	2012	2013	Total 2003–13	Change 2003-13
**Bilateral aid agencies**	2855.8	2614.6	3849.5	4037.5	4924.1	5401.1	6529.4	6881.3	7433.5	7654.3	8285.0	60466.2	190%
Australia	111.3	101.6	103.0	128.2	126.8	156.0	154.8	187.4	231.7	325.3	217.7	1843.9	96%
Austria	4.6	5.7	6.2	9.5	7.6	8.0	7.3	6.4	7.9	4.6	4.5	72.2	-2%
Belgium	46.4	45.6	51.1	54.4	70.7	54.2	58.8	68.1	67.5	53.3	62.0	632.0	34%
Canada	138.2	181.7	200.0	207.4	354.8	286.1	329.2	286.1	471.1	498.3	560.4	3513.1	305%
Czech Republic									1.1	2.4	1.8	5.3	
Denmark	34.3	36.6	44.9	41.3	46.1	52.0	76.2	98.9	91.0	69.4	63.0	653.7	84%
Estonia											0.4	0.4	
Finland	15.6	0.0	0.0	19.5	25.1	30.4	26.0	29.3	23.8	25.4	23.7	218.8	52%
France	80.5	25.7	29.3	15.7	27.8	64.9	154.4	94.9	83.8	102.0	126.1	805.2	57%
Germany	141.4	136.2	128.3	163.1	202.8	231.1	251.2	283.3	211.5	279.9	324.7	2353.6	130%
Greece	29.3	17.0	14.9	16.6	16.5	5.6	10.5	13.0	0.8	0.7	0.1	125.0	-100%
Iceland									1.1	1.2	1.8	4.2	
Ireland	44.5	44.7	51.7	65.8	96.8	73.1	69.2	60.3	65.3	52.3	50.3	673.8	13%
Italy	28.7	36.2	44.0	36.7	49.2	61.8	47.5	44.0	39.6	29.6	39.4	456.9	37%
Japan	117.4	96.7	94.9	176.6	211.1	152.2	205.8	208.6	204.1	254.4	254.9	1976.6	117%
Korea	0.0			14.3	16.3	29.2	49.7	57.8	37.1	50.3	62.4	317.1	
Kuwait (KFAED)								8.6	6.8	1.7	1.3	18.4	
Luxembourg	0.0	18.4	20.3	23.7	35.7	33.8	26.9	30.6	16.6	18.1	24.5	248.6	
Netherlands	145.7	122.8	117.8	154.3	158.7	196.8	220.7	202.6	174.0	174.7	195.3	1863.5	34%
New Zealand	5.7	10.2	10.4	15.0	7.1	13.8	13.9	16.8	18.0	17.1	13.8	141.8	139%
Norway	68.7	140.9	271.0	125.4	139.7	146.1	196.4	148.6	147.9	143.4	225.4	1753.6	228%
Poland											0.6	0.6	
Portugal	3.1	3.9	6.7	6.0	3.8	3.1	3.1	5.4	5.5	5.2	5.7	51.4	83%
Slovak Republic											0.3	0.3	
Slovenia								0.2	0.2	0.6	0.4	1.3	
Spain	56.9	57.7	94.0	93.5	102.3	204.7	180.4	180.2	64.1	39.2	38.4	1111.2	-32%
Sweden	70.9	112.1	145.0	149.2	148.5	141.4	161.1	157.0	113.1	245.1	173.6	1613.2	145%
Switzerland	29.5	26.1	31.6	32.0	28.4	36.4	41.0	37.7	41.8	45.6	51.1	401.2	74%
United Arab Emirates							38.9	20.9	16.3	52.4	68.0	196.5	
United Kingdom	320.1	291.1	452.4	464.6	472.1	543.6	728.9	716.1	948.4	984.1	1350.5	7271.8	322%
United States	1362.9	1103.8	1932.1	2024.5	2576.3	2876.8	3477.9	3918.5	4347.2	4178.0	4342.8	32140.8	219%
**Multilateral aid agencies**	**1106.2**	**1212.5**	**1371.8**	**1451.2**	**1225.8**	**1329.5**	**1855.4**	**1698.6**	**1921.9**	**1671.7**	**1822.8**	**16667.5**	**65%**
African Development Bank	0.0	0.0	0.0	0.0	0.0	0.0	0.0	0.0	0.3	0.1	0.2	0.6	
African Development Fund	15.5	31.3	27.3	33.5	39.7	55.5	60.0	48.0	43.4	40.8	23.1	418.1	50%
AFESD						1.1	0.4	0.9	1.7	1.6	1.4	7.1	
Asian Development Bank Special Funds	0.0	0.0	0.0	0.0	0.0	0.0	0.0	88.2	40.7	45.5	27.0	201.4	
BADEA									2.9	1.0	0.7	4.6	
European Union Institutions	80.7	105.0	234.5	371.0	297.7	359.0	402.2	429.9	528.6	516.2	532.7	3857.5	560%
Global Environment Facility	0.0	0.0	0.0	0.0	0.0	0.3	0.0	0.0	0.0	0.1	0.4	0.8	
IDA	468.6	697.6	697.2	600.4	428.9	412.1	634.8	446.4	568.6	439.0	565.6	5958.7	21%
IDB Special Fund	0.0	0.0	0.0	0.0	0.0	0.0	11.3	14.7	32.1	22.2	35.1	115.4	

TRIAL MODE - a valid license will remove this message. See the keywords property of this PDF for more information.

International Monetary Fund CTF	37.2	46.6	21.6	32.0	15.6	45.6	99.1	51.8	45.6	41.0	33.3	469.6	-11%
OFID							11.4	11.5	10.1	12.5	11.7	57.2	
UNAIDS	81.5	80.2	69.0	89.0	67.8	61.8	81.1	102.1	118.2	58.1	50.1	859.0	-39%
UNDP		8.6	10.4	11.2	7.4	8.0	11.3	8.4	7.9	11.8	7.3	92.3	
UNFPA	326.8	139.7	136.6	130.7	126.4	143.7	126.1	104.8	151.0	153.9	150.3	1689.8	-54%
UNHCR									0.0	0.0	0.0	0.0	
UNICEF	95.9	103.5	138.9	145.0	201.3	201.0	185.9	185.8	161.4	130.8	165.2	1714.7	72%
UNPBF					0.0	0.0	0.1	0.1	0.4	0.2	0.0	0.8	
UNRWA			36.4	38.6	40.9	41.6	44.2	49.2	47.9	54.6	42.9	396.3	
WFP						0.0	38.4	32.9	12.5	13.3	23.1	120.2	
WHO							149.2	124.0	148.5	129.1	152.7	703.4	
**Global health initiatives**	**344.8**	**494.8**	**847.5**	**968.3**	**1712.3**	**1719.1**	**1655.2**	**2172.6**	**1867.1**	**2592.8**	**3214.2**	**17588.7**	**832%**
GAVI	212.1	167.3	259.2	245.4	876.2	640.7	423.8	664.7	691.4	999.5	1371.4	6551.5	546%
Global Fund	132.7	327.5	588.3	722.9	836.1	1078.4	1231.4	1507.9	1175.8	1593.4	1842.8	11037.2	1289%
**Private donor**							**537.3**	**546.1**	**717.4**	**669.6**	**667.0**	**3137.4**	
Bill & Melinda Gates Foundation							537.3	546.1	717.4	669.6	667.0	3137.4	
**Grand Total**	**4306.8**	**4322.0**	**6068.8**	**6457.0**	**7862.2**	**8449.7**	**10577.3**	**11298.7**	**11939.9**	**12588.4**	**13988.9**	**97859.8**	**225%**

AFESD = Arab Fund for Economic and Social Development; BADEA = Arab Bank for Economic Development in Africa; IDA = International Development Association; IDB = Inter-American Development Bank; CTF = Concessional Trust Funds; OFID = Organization of the Petroleum Exporting Countries Fund for International Development; UNAIDS = Joint United Nations Programme on HIV/AIDS; UNDP = United Nations Development Programme; UNFPA = United Nations Population Fund; UNHCR = United Nations High Commissioner For Refugees; UNICEF = United Nations Children's Fund; UNPBF = United Nations Peacebuilding Fund; UNRWA = United Nations Relief and Works Agency for Palestine Refugees in the Near East; WFP = World Food Programme; WHO = World Health Organization; GAVI = Global Alliance for Vaccines and Immunization/the Vaccine Alliance.

TRIAL MODE - a valid license will remove this message. See the keywords property of this PDF for more information.

TRIAL MODE - a valid license will remove this message. See the keywords property of this PDF for more information.

Appendix Table 6: ODA+ for reproductive, maternal, newborn and child health to 75 priority recipient countries, 2003–13, $m 2013

	2003	2004	2005	2006	2007	2008	2009	2010	2011	2012	2013	Total	2003–13
Afghanistan	50·1	61·6	112·2	115·1	208·1	258·5	409·4	368·8	407·6	363·3	377·0	2731·2	654%
Angola	40·8	42·3	90·9	58·1	89·9	103·7	74·5	83·9	65·2	88·3	98·7	836·3	142%
Azerbaijan	3·4	2·2	5·6	5·9	5·6	7·9	10·3	13·2	15·2	10·6	13·9	93·7	313%
Bangladesh	179·8	124·7	237·2	312·2	110·4	185·8	305·4	267·0	261·9	259·7	412·4	2656·6	129%
Benin	28·6	35·7	41·5	40·1	49·4	58·8	83·3	90·5	95·7	70·3	80·2	674·2	180%
Bolivia	57·4	30·5	29·1	34·3	50·1	52·0	55·3	52·3	51·2	51·4	45·3	509·0	-21%
Botswana	17·4	28·4	17·0	23·9	38·0	124·8	139·5	59·5	62·8	39·8	51·6	602·7	196%
Brazil	19·9	11·4	12·6	12·3	7·5	10·5	15·4	17·4	14·1	6·3	5·1	132·6	-74%
Burkina Faso	36·6	38·6	80·6	62·0	78·3	100·6	124·2	146·8	84·9	131·4	136·4	1020·5	273%
Burundi	21·8	24·1	30·2	40·6	45·4	58·5	67·7	83·2	81·1	67·3	93·5	613·3	329%
Cambodia	69·7	55·0	74·7	73·2	81·8	92·0	118·7	151·5	150·0	125·5	132·6	1124·8	90%
Cameroon	26·7	32·8	44·1	41·6	51·0	42·7	67·6	48·8	113·0	73·3	97·8	639·2	267%
Central African Republic	8·7	12·2	14·3	14·5	20·3	28·6	20·9	26·1	28·2	26·4	37·7	237·8	331%
Chad	24·2	24·9	36·9	23·2	29·9	40·0	44·1	76·4	54·6	59·3	105·6	519·2	336%
China	83·9	93·0	104·1	127·4	173·8	133·9	135·7	105·1	63·9	65·0	41·0	1126·8	-51%
Comoros	4·6	3·0	1·6	1·5	1·7	1·7	2·4	7·9	4·9	7·6	9·0	45·8	97%
Congo, Rep.	6·5	8·4	8·8	10·5	13·2	17·5	10·0	34·2	26·1	16·0	18·5	169·7	187%
Cote d'Ivoire	41·6	30·7	34·5	38·6	60·8	80·3	100·8	141·8	110·3	103·4	133·8	876·7	222%
DPR Korea	3·9	8·3	10·5	6·7	11·2	8·7	19·8	21·8	10·4	18·0	24·5	143·8	536%
DR Congo	77·3	82·5	126·8	152·7	170·9	286·9	360·8	398·7	434·3	475·5	549·6	3112·3	611%
Djibouti	3·0	5·3	10·1	11·5	22·4	12·8	13·9	7·9	8·9	14·8	9·9	120·4	230%
Egypt	47·8	50·8	68·1	86·9	70·3	83·2	42·1	45·9	26·9	21·8	14·8	558·5	-69%
Equatorial Guinea	2·7	2·0	4·8	7·8	5·1	11·4	8·3	8·8	3·5	1·6	0·5	56·6	-80%
Eritrea	33·6	34·0	35·1	23·4	31·4	30·7	27·0	47·0	19·6	26·1	33·1	341·0	-2%
Ethiopia	171·2	138·9	208·6	366·9	509·3	449·8	510·3	614·6	753·7	655·6	872·3	5251·2	409%
Gabon	2·7	4·5	5·5	7·0	7·4	5·0	8·5	3·7	4·4	3·9	5·1	57·6	91%
Gambia	11·2	14·3	20·6	9·2	14·1	11·0	16·1	19·4	17·5	17·0	28·2	178·5	152%
Ghana	79·8	110·8	123·9	114·0	133·3	132·8	219·7	200·9	201·2	250·8	257·7	1824·7	223%

TRIAL MODE - a valid license will remove this message. See the keywords property of this PDF for more information.

Guatemala	27·1	18·2	25·7	38·6	40·3	58·7	45·3	35·3	51·0	45·4	41·0	426·5	51%
Guinea	21·1	21·4	29·0	30·1	25·8	32·6	30·9	45·0	37·1	50·5	41·5	365·2	97%
Guinea-Bissau	4·7	3·6	8·1	8·9	13·7	11·1	13·5	19·8	13·0	6·0	27·2	129·7	478%
Haiti	45·1	56·3	84·1	137·8	151·3	127·2	163·0	254·7	282·0	200·7	197·2	1699·4	338%
India	373·4	489·4	536·0	348·0	521·2	509·2	614·6	576·5	783·0	602·4	495·4	5849·1	33%
Indonesia	113·8	111·4	139·1	141·4	121·4	136·9	132·4	158·8	126·1	156·5	177·3	1514·8	56%
Iraq	84·0	52·3	246·6	141·2	146·8	40·7	57·2	46·0	16·9	26·2	28·5	886·5	-66%
Kenya	162·7	161·8	178·3	241·4	275·2	296·4	445·5	546·7	642·6	686·6	755·8	4392·9	364%
Kyrgyz Republic	28·7	10·6	16·7	15·3	17·8	24·1	21·7	22·4	25·5	19·1	18·8	220·7	-34%
Lao PDR	17·5	14·0	18·7	16·6	26·7	27·0	26·8	37·3	37·7	40·9	45·8	309·1	161%
Lesotho	10·1	10·2	8·6	9·9	21·2	25·8	30·8	51·4	73·5	73·5	84·2	399·3	736%
Liberia	11·3	14·5	14·9	25·1	33·4	80·0	78·3	79·0	94·6	98·8	94·5	624·4	739%
Madagascar	57·3	44·6	50·4	49·4	78·2	90·2	76·1	131·9	80·9	92·2	143·1	894·2	150%
Malawi	105·5	100·4	116·1	132·2	179·0	194·9	207·0	181·3	251·6	327·0	333·3	2128·4	216%
Mali	37·7	41·5	64·4	70·2	94·3	98·9	120·1	133·2	178·6	174·9	199·6	1213·4	430%
Mauritania	12·3	12·8	7·7	10·0	19·2	17·6	16·5	17·0	15·5	17·6	17·5	163·6	43%
Mexico	10·2	6·5	4·5	5·0	7·0	3·7	3·5	4·4	5·6	7·3	5·5	63·1	-46%
Morocco	29·0	12·7	17·5	28·2	29·0	21·3	40·9	38·2	27·9	29·0	48·7	322·5	68%
Mozambique	136·3	160·4	166·9	193·6	272·6	283·1	307·6	367·6	391·7	398·4	433·7	3111·8	218%
Myanmar	24·3	23·1	37·3	29·3	27·0	53·4	46·7	58·9	52·1	132·6	153·5	638·1	533%
Nepal	51·4	42·5	52·4	69·3	59·4	76·4	75·8	103·0	82·0	78·8	87·1	778·1	69%
Niger	23·2	21·9	47·9	56·6	66·6	88·4	95·2	114·2	106·6	89·0	107·7	817·2	365%
Nigeria	139·6	174·7	209·6	315·0	416·8	478·2	875·4	570·2	743·6	859·6	1211·5	5994·3	768%
Pakistan	111·8	70·1	188·8	208·6	294·6	215·7	349·5	452·0	461·8	490·6	554·8	3398·3	396%
Papua New Guinea	62·1	51·7	48·3	65·8	72·0	84·0	95·9	76·9	114·1	104·8	126·8	902·3	104%
Peru	24·0	15·5	41·4	30·4	34·0	37·2	96·8	46·0	31·0	57·0	46·6	459·9	94%
Philippines	61·9	48·9	76·2	69·5	72·0	57·5	69·4	81·0	73·1	70·0	82·0	761·4	32%
Rwanda	46·5	58·7	76·1	124·7	131·4	169·5	211·2	230·0	260·3	246·4	217·0	1771·7	366%
Sao Tome and Principe	2·3	2·2	4·6	4·0	4·1	5·5	3·0	3·6	6·9	3·7	8·7	48·7	273%
Senegal	67·4	70·5	67·6	52·9	61·5	82·8	103·1	88·7	112·0	127·4	121·4	955·1	80%
Sierra Leone	16·6	20·6	32·0	29·4	36·6	46·0	70·9	73·8	74·1	64·6	95·0	559·6	472%

TRIAL MODE - a valid license will remove this message. See the keywords property of this PDF for more information.

Solomon Islands	7·8	7·5	6·7	6·6	7·5	11·4	14·7	15·2	16·6	10·2	16·8	121·2	116%
Somalia	9·8	16·7	21·5	43·0	44·2	43·4	69·3	63·7	64·1	118·4	95·7	589·8	878%
South Africa	82·4	87·0	134·1	158·9	263·0	291·2	417·4	454·7	531·9	507·8	478·0	3406·6	480%
South Sudan									87·1	197·8	206·6	491·4	
Sudan	19·3	62·5	142·6	108·4	134·1	168·2	154·4	231·6	114·1	144·7	160·9	1440·6	736%
Swaziland	9·6	4·3	20·8	12·8	18·1	16·5	27·8	49·6	58·8	46·1	52·1	316·6	441%
Tajikistan	7·4	10·2	11·4	13·2	16·6	20·5	22·0	36·2	19·5	29·2	29·7	215·8	303%
Tanzania	129·9	160·6	213·8	249·8	327·8	413·4	462·1	556·3	560·9	599·5	750·1	4424·3	478%
Togo	10·8	11·1	14·8	11·5	21·9	26·5	36·2	33·1	42·0	12·9	46·3	267·0	330%
Turkmenistan	2·4	1·7	2·3	2·0	3·4	2·2	1·7	2·0	1·9	1·5	1·9	23·0	-18%
Uganda	153·2	188·9	256·7	232·1	261·3	234·5	316·9	356·3	390·6	520·1	455·2	3365·8	197%
Uzbekistan	17·9	14·5	13·4	15·1	16·0	20·2	26·3	27·1	22·1	31·7	24·6	228·9	37%
Vietnam	84·1	57·4	76·2	90·2	83·8	99·8	102·6	125·8	142·5	123·0	151·9	1137·4	81%
Yemen	26·7	18·1	58·8	43·6	51·1	53·5	49·8	65·2	55·0	112·4	202·4	736·6	659%
Zambia	118·6	165·1	172·4	160·9	221·2	242·0	262·4	225·6	341·0	338·8	422·4	2670·4	256%
Zimbabwe	53·0	45·5	79·5	63·3	133·7	87·2	143·2	183·3	164·4	396·2	274·8	1624·1	419%
**Total**	**4306·8**	**4322·0**	**6068·8**	**6457·0**	**7862·2**	**8449·7**	**10577·3**	**11298·7**	**11939·9**	**12588·4**	**13988·9**	**97859·8**	**225%**

TRIAL MODE - a valid license will remove this message. See the keywords property of this PDF for more information.

Appendix Table 7: Funding to child health per child under 5, 2013 US$

	2003	2004	2005	2006	2007	2008	2009	2010	2011	2012	2013
Afghanistan	6·06	9·22	15·85	16·26	24·52	30·54	37·34	35·88	38·23	36·71	42·70
Angola	5·95	5·93	15·48	8·85	12·57	15·24	9·50	10·53	7·67	11·89	10·29
Azerbaijan	2·55	1·91	5·95	2·58	2·61	2·91	4·08	6·37	7·41	4·76	8·39
Bangladesh	2·34	2·40	4·62	7·46	2·61	5·74	7·58	8·25	8·03	8·17	14·03
Benin	6·79	7·70	13·05	11·49	15·27	19·02	21·77	23·66	25·73	22·28	24·23
Bolivia	17·01	11·87	12·96	13·91	18·28	14·85	14·19	16·14	12·62	16·39	13·61
Botswana	4·45	2·55	8·27	4·57	6·74	14·55	14·67	18·52	8·29	6·17	11·07
Brazil	0·26	0·14	0·13	0·18	0·12	0·27	0·30	0·34	0·34	0·06	0·08
Burkina Faso	5·32	6·91	13·23	9·33	11·74	14·56	18·37	24·62	13·17	20·02	18·11
Burundi	8·31	8·49	9·77	13·56	13·86	18·46	14·39	23·53	20·47	16·15	21·20
Cambodia	10·23	6·61	14·28	9·93	12·88	14·23	21·30	36·50	23·76	26·26	22·31
Cameroon	3·04	3·99	7·13	5·53	4·26	5·87	8·36	6·45	18·50	10·02	11·46
Central African Republic	3·02	8·88	9·39	7·44	12·47	16·89	15·60	15·39	20·00	19·44	29·10
Chad	3·49	4·29	7·27	3·23	6·58	9·75	9·41	17·36	11·30	14·22	23·12
China (People's Republic of)	0·47	0·55	0·57	0·55	0·68	0·49	0·37	0·50	0·25	0·31	0·14
Comoros	28·10	17·66	9·94	6·02	5·51	5·75	9·64	38·09	19·62	27·02	35·62
Congo	6·23	7·05	5·44	4·05	7·89	10·53	5·65	24·90	16·15	11·02	13·03
Côte d'Ivoire	3·24	2·79	3·52	3·44	6·04	6·82	12·10	18·09	12·08	8·85	14·05
Democratic People's Republic of Korea	1·48	3·20	3·66	2·15	2·75	2·68	5·32	6·93	3·46	5·88	7·11
Democratic Republic of the Congo	3·37	4·52	5·82	7·65	7·43	13·67	16·12	17·27	19·38	21·53	26·88
Djibouti	11·58	26·83	38·32	41·02	91·68	50·40	56·06	28·92	28·27	57·04	38·64
Egypt	0·94	1·02	2·50	3·73	2·03	3·36	1·12	1·95	0·94	1·22	0·60
Equatorial Guinea	14·00	12·35	21·48	41·93	25·05	52·89	37·13	42·32	15·34	6·32	2·03
Eritrea	18·06	15·74	14·06	9·48	10·54	10·01	8·12	19·77	7·02	7·80	12·98
Ethiopia	5·11	3·28	5·64	11·77	12·97	11·44	14·62	14·04	19·65	19·17	27·24
Gabon	4·65	9·57	15·11	15·67	11·03	7·42	12·41	3·76	4·42	3·97	5·28
Gambia	9·99	14·77	26·90	12·23	22·86	18·22	21·29	30·37	25·33	26·19	42·11
Ghana	10·92	17·13	19·79	16·42	15·69	15·88	25·02	24·65	22·24	31·98	31·65
Guatemala	5·13	1·22	4·52	7·87	5·35	8·03	5·88	4·10	5·41	5·05	3·60

TRIAL MODE - a valid license will remove this message. See the keywords property of this PDF for more information.

Guinea	2·47	3·01	5·48	6·10	4·25	5·77	5·60	10·01	4·01	10·91	6·78
Guinea-Bissau	6·69	7·11	14·55	13·99	21·84	20·77	18·27	35·00	15·59	6·37	53·68
Haiti	3·22	8·87	6·82	13·50	14·71	19·30	20·09	53·79	53·68	26·44	30·47
India	1·51	2·19	2·08	1·45	1·83	2·08	1·81	1·97	1·98	2·11	1·40
Indonesia	1·84	1·35	2·39	2·32	2·13	2·69	2·24	2·41	1·58	2·53	3·07
Iraq	12·35	8·40	35·03	16·97	16·57	5·75	6·94	6·05	2·06	2·54	2·84
Kenya	7·64	6·52	11·88	14·22	10·26	12·08	16·24	22·38	24·47	23·53	25·19
Kyrgyzstan	9·64	9·58	16·63	15·53	14·53	22·32	19·28	18·86	18·92	13·25	12·68
Lao People's Democratic Republic	12·86	10·78	12·71	11·44	15·31	18·03	13·30	22·34	19·53	20·87	24·37
Lesotho	6·86	9·70	5·65	6·37	13·83	12·88	15·00	40·92	51·75	48·48	103·39
Liberia	13·23	15·36	12·64	23·54	19·77	63·95	54·30	52·25	58·80	54·86	43·44
Madagascar	6·17	5·43	8·89	7·18	10·95	13·34	10·87	20·06	10·46	12·54	21·11
Malawi	14·32	9·76	19·08	18·80	21·97	28·82	30·11	25·46	31·86	41·19	42·59
Mali	6·53	7·38	11·71	12·16	14·40	15·00	18·42	23·57	27·74	24·20	31·07
Mauritania	7·93	8·84	5·31	4·35	10·38	12·88	14·39	15·20	10·92	12·11	13·19
Mexico	0·17	0·11	0·12	0·17	0·21	0·09	0·12	0·07	0·06	0·11	0·07
Morocco	1·54	0·93	2·15	3·97	2·02	2·05	5·17	5·34	3·70	3·56	5·96
Mozambique	12·34	14·21	15·15	12·66	16·80	20·98	23·01	27·50	26·14	30·07	30·94
Myanmar	2·31	2·32	4·06	3·24	2·64	6·31	4·68	5·81	4·94	14·52	14·86
Nepal	3·49	3·60	4·82	3·99	5·73	7·80	6·95	14·20	10·73	9·88	9·01
Niger	4·57	4·04	9·14	10·34	10·11	15·24	13·87	18·25	14·15	9·90	15·90
Nigeria	2·30	3·67	3·60	5·13	5·18	6·80	13·49	6·63	10·34	12·17	18·24
Pakistan	2·92	2·04	6·19	7·38	6·09	5·54	9·01	12·09	13·04	14·18	14·41
Papua New Guinea	32·37	26·16	23·22	18·34	20·54	23·12	32·01	23·02	33·87	35·11	35·58
Peru	2·10	2·21	6·12	4·02	4·00	4·41	16·61	4·90	2·90	10·15	9·44
Philippines	1·10	1·15	0·55	0·92	1·62	1·00	1·28	2·53	1·59	1·29	1·54
Rwanda	10·30	11·71	16·16	24·43	17·05	30·35	39·90	28·66	32·37	33·35	33·36
Sao Tome and Principe	49·15	45·98	111·36	89·41	76·10	113·87	53·19	71·56	108·85	65·20	130·05
Senegal	17·19	17·19	14·78	10·91	12·48	18·77	19·19	16·36	18·43	26·52	21·27
Sierra Leone	8·31	11·77	14·74	14·61	15·29	18·18	27·88	34·09	29·22	24·76	36·21
Solomon Islands	52·81	59·69	47·02	41·61	41·52	84·78	94·22	88·99	88·64	66·61	107·36
Somalia	3·04	6·35	7·31	11·95	11·34	12·48	20·17	18·18	15·36	24·86	22·58
South Africa	2·48	1·34	4·10	3·92	3·98	3·22	3·95	3·04	6·84	6·32	8·74

TRIAL MODE - a valid license will remove this message. See the keywords property of this PDF for more information.

South Sudan									5·56	13·00	43·74
Sudan	2·07	7·26	15·89	10·19	13·02	17·28	14·55	19·69	10·51	15·48	17·09
Swaziland	6·27	2·18	7·09	8·16	12·20	9·62	30·08	22·66	33·64	18·85	28·17
Tajikistan	3·61	6·27	7·29	8·57	7·62	12·15	11·41	15·89	7·84	10·29	9·22
Tanzania	5·93	8·74	10·68	11·29	12·75	18·94	19·77	22·47	18·74	22·47	28·58
Togo	3·51	4·95	7·51	3·74	8·13	13·93	15·27	14·58	18·67	4·17	21·17
Turkmenistan	1·74	1·58	2·82	1·91	3·16	1·33	1·01	2·42	1·65	0·76	1·57
Uganda	10·15	9·35	14·52	13·61	11·30	12·24	14·03	13·77	12·60	19·88	15·63
Uzbekistan	1·98	3·38	2·57	2·75	2·44	3·83	4·80	5·60	3·86	5·19	4·59
Viet Nam	2·46	2·98	5·23	5·51	2·90	5·59	4·16	7·49	7·38	7·59	9·92
Yemen	4·86	2·32	9·28	5·12	6·54	6·52	4·53	8·15	8·97	14·84	21·64
Zambia	19·88	29·06	25·74	22·97	29·15	29·44	31·55	22·22	30·37	36·62	43·62
Zimbabwe	6·15	4·67	10·12	9·83	15·50	15·32	23·22	26·14	21·18	57·37	35·00

TRIAL MODE - a valid license will remove this message. See the keywords property of this PDF for more information.

Appendix Table 8: Funding to maternal and newborn health per live birth, 2013 US$

	2003	2004	2005	2006	2007	2008	2009	2010	2011	2012	2013
Afghanistan	10·75	10·25	14·78	17·62	29·02	38·77	66·99	66·36	91·33	77·73	84·05
Angola	7·98	8·90	17·50	7·30	9·17	12·10	12·18	7·79	4·75	9·07	17·85
Azerbaijan	6·81	4·91	4·74	5·74	4·79	5·62	8·40	9·52	16·24	15·62	10·48
Bangladesh	10·82	16·09	15·63	32·61	8·59	13·04	30·04	19·55	18·73	20·16	28·00
Benin	17·66	22·33	10·97	12·03	18·35	21·60	37·55	36·48	36·18	27·93	34·93
Bolivia	69·45	29·84	22·62	24·63	30·36	28·61	30·34	37·65	44·80	33·98	28·90
Botswana	38·18	12·16	12·98	9·58	14·25	14·13	10·58	8·86	33·43	12·71	15·68
Brazil	0·65	0·34	0·45	0·62	0·31	0·35	0·59	0·74	0·68	0·43	0·58
Burkina Faso	11·40	12·27	32·89	15·03	16·08	23·30	23·42	30·54	22·90	42·25	36·91
Burundi	10·88	12·96	14·59	12·12	17·56	21·43	24·92	35·95	25·91	25·03	32·01
Cambodia	20·45	13·84	26·87	22·64	28·17	48·82	62·12	83·25	52·00	74·89	72·16
Cameroon	5·17	5·06	5·60	7·33	6·14	6·25	8·80	7·84	17·32	11·62	12·76
Central African Republic	12·83	10·52	10·21	12·79	27·95	23·09	18·49	22·95	19·33	29·38	38·02
Chad	13·14	15·28	14·63	11·94	8·51	11·22	11·72	20·24	9·24	16·63	32·42
China (People's Republic of)	0·88	1·12	0·89	1·29	1·48	1·18	0·93	0·98	0·53	0·66	0·49
Comoros	29·99	20·11	12·85	15·33	17·13	21·45	23·55	61·69	38·38	93·64	99·68
Congo	8·59	9·72	8·29	6·97	6·32	14·10	3·60	24·36	21·62	14·94	8·85
Côte d'Ivoire	5·97	4·45	2·43	4·44	5·97	7·95	13·75	17·25	12·74	18·46	23·53
Democratic People's Republic of Korea	1·20	3·65	5·20	4·58	9·00	6·50	16·75	14·57	6·09	10·70	17·04
Democratic Republic of the Congo	3·79	4·72	7·35	8·93	7·40	14·89	17·72	15·37	18·65	26·29	25·02
Djibouti	25·52	65·06	75·36	94·93	148·56	81·51	130·00	63·62	73·14	199·97	43·49
Egypt	6·85	2·38	7·26	8·36	5·45	6·24	4·57	4·14	3·98	2·49	2·27
Equatorial Guinea	32·41	21·42	28·66	63·38	40·43	95·22	75·77	60·42	24·48	18·46	5·79
Eritrea	40·73	37·20	28·35	21·98	27·86	24·65	15·94	31·17	16·20	15·06	27·44
Ethiopia	7·03	6·00	6·53	19·54	17·70	16·90	22·28	18·50	26·11	30·91	34·73
Gabon	11·92	15·51	18·60	20·77	19·67	15·18	14·01	7·32	11·60	12·37	16·38
Gambia	61·52	61·37	58·77	21·06	25·60	23·00	27·85	29·56	21·08	25·43	33·21
Ghana	22·00	26·61	27·33	25·03	25·40	34·10	50·80	54·99	41·49	65·08	46·15
Guatemala	12·99	17·50	15·93	24·20	25·07	31·43	18·23	15·80	23·01	25·40	17·94

TRIAL MODE - a valid license will remove this message. See the keywords property of this PDF for more information.

Guinea	7·95	8·29	13·50	13·00	10·87	13·19	11·46	19·34	20·27	25·94	16·44
Guinea-Bissau	32·97	18·06	28·06	30·04	39·91	33·96	38·86	47·61	40·86	31·67	102·63
Haiti	6·39	15·31	19·82	34·26	42·08	60·86	55·39	147·45	187·27	128·60	111·95
India	1·93	3·75	4·60	2·41	3·03	3·98	5·22	4·13	8·05	4·93	4·07
Indonesia	4·41	6·92	5·41	7·24	5·35	6·67	4·87	9·53	7·41	8·51	7·37
Iraq	19·99	12·11	60·31	46·27	35·53	7·52	12·00	8·13	3·19	7·47	4·74
Kenya	14·68	10·72	16·42	18·88	18·52	14·72	29·55	28·65	26·79	26·96	34·99
Kyrgyzstan	144·69	30·03	35·70	26·73	24·88	28·46	27·59	34·19	32·87	28·76	25·71
Lao People's Democratic Republic	21·31	18·38	23·26	21·47	30·65	30·48	33·94	39·76	39·88	67·38	51·30
Lesotho	22·96	28·12	10·71	11·86	23·94	15·50	23·88	56·00	99·50	80·63	123·31
Liberia	9·20	18·89	15·25	32·98	52·94	112·83	104·22	88·67	119·94	195·25	186·62
Madagascar	18·40	12·89	12·67	13·75	19·92	27·90	17·84	28·00	23·08	17·40	27·42
Malawi	30·33	18·91	18·28	29·94	28·76	48·62	48·09	39·69	41·40	74·11	82·89
Mali	18·99	19·86	28·05	25·44	26·04	29·52	34·68	31·37	56·96	64·98	61·43
Mauritania	39·39	31·83	13·00	14·14	42·86	26·44	31·36	36·46	32·32	40·00	32·16
Mexico	1·37	0·59	0·56	0·59	0·65	0·39	0·20	0·33	0·25	0·37	0·24
Morocco	21·10	9·00	7·64	12·92	18·12	13·23	18·68	14·52	6·37	12·35	19·75
Mozambique	27·97	22·01	24·10	19·70	33·27	31·58	30·33	34·99	46·53	51·64	64·01
Myanmar	4·66	5·53	5·44	7·13	4·45	11·61	11·09	11·26	10·38	35·73	42·77
Nepal	10·41	7·36	8·25	24·94	20·47	40·57	33·12	35·41	27·03	26·56	26·99
Niger	6·98	5·98	14·08	14·58	18·01	21·41	21·14	30·75	27·65	34·10	18·70
Nigeria	2·12	1·25	2·15	2·72	4·66	4·42	13·65	6·35	8·06	11·54	11·76
Pakistan	3·50	3·58	7·24	5·35	4·98	12·23	12·22	16·48	13·65	15·65	21·03
Papua New Guinea	67·55	56·66	32·93	43·58	23·83	34·31	48·37	41·56	101·68	50·94	122·08
Peru	14·18	6·72	15·54	11·45	9·90	11·34	16·47	15·08	10·35	11·33	7·16
Philippines	3·49	2·37	2·80	3·38	4·82	5·79	5·11	6·77	4·44	7·92	9·66
Rwanda	15·17	16·09	19·69	35·54	23·67	43·03	61·35	42·14	49·60	42·19	42·20
Sao Tome and Principe	112·97	127·46	171·04	134·74	106·34	158·46	96·61	79·25	220·50	107·93	350·58
Senegal	25·51	26·23	29·99	15·87	14·66	28·98	37·12	24·84	34·30	48·57	30·83
Sierra Leone	15·31	21·71	29·09	29·96	32·86	58·32	82·56	60·78	82·41	60·55	127·17
Solomon Islands	140·52	144·40	106·57	109·21	105·27	117·77	157·19	179·14	250·05	126·36	224·25
Somalia	5·30	8·56	6·07	26·01	17·59	23·85	32·38	22·64	31·27	94·01	55·36
South Africa	5·30	4·21	3·78	3·87	3·00	3·72	3·74	0·81	5·27	8·77	11·99

South Sudan									13·02	46·80	136·14
Sudan	3·19	11·23	20·22	14·78	14·12	22·80	20·15	34·53	13·15	15·89	15·78
Swaziland	4·36	5·60	5·94	5·42	7·23	11·51	38·95	18·80	76·54	21·07	33·13
Tajikistan	8·42	10·30	10·76	10·92	10·86	11·15	14·59	24·50	13·68	20·15	28·97
Tanzania	13·17	11·03	14·61	18·85	17·52	25·54	26·09	28·19	36·03	35·36	50·39
Togo	8·26	7·13	6·74	4·79	10·26	19·04	12·90	21·45	29·87	9·41	26·42
Turkmenistan	6·13	5·54	2·85	4·52	5·14	4·80	3·45	1·63	2·61	4·31	4·16
Uganda	18·70	11·64	40·16	15·76	12·22	13·18	13·87	16·66	17·85	29·74	22·84
Uzbekistan	3·54	4·21	3·36	4·46	4·21	7·06	6·00	5·67	4·89	6·09	7·10
Viet Nam	14·95	9·93	10·19	11·19	9·96	12·35	16·53	13·98	16·23	14·67	15·45
Yemen	6·13	7·51	18·26	21·03	18·10	26·11	21·39	22·02	12·87	47·85	79·25
Zambia	27·85	36·88	26·87	26·80	38·42	34·35	41·44	23·24	55·48	48·13	68·42
Zimbabwe	9·45	13·44	22·08	15·93	23·94	27·36	42·09	47·50	48·13	173·02	103·64

TRIAL MODE - a valid license will remove this message. See the keywords property of this PDF for more information.

TRIAL MODE - a valid license will remove this message. See the keywords property of this PDF for more information.

Appendix Table 9: Funding to reproductive and sexual health per woman aged 15–49, 2013 US$

	2003	2004	2005	2006	2007	2008	2009	2010	2011	2012	2013
Afghanistan	0·15	0·02	0·19	0·04	0·06	3·01	8·42	5·64	5·20	5·12	1·68
Angola	1·86	2·60	2·68	2·76	3·09	4·14	2·19	4·14	3·40	3·18	4·07
Azerbaijan	0·01	0·01	0·16	0·71	0·59	1·16	1·16	1·29	0·97	0·56	0·85
Bangladesh	1·53	0·31	1·54	0·77	0·34	0·46	0·41	0·51	0·51	0·52	0·79
Benin	3·36	6·17	5·96	5·51	3·06	4·81	6·77	8·81	7·68	3·78	4·06
Bolivia	1·55	1·29	0·21	1·43	1·14	4·94	5·43	3·00	3·17	3·03	3·18
Botswana	18·35	44·26	20·92	31·67	50·66	175·59	190·85	80·11	78·79	53·55	68·82
Brazil	0·15	0·11	0·11	0·09	0·04	0·04	0·08	0·10	0·05	0·04	0·02
Burkina Faso	2·62	2·66	3·78	4·86	4·62	7·31	7·56	6·83	3·02	4·32	7·08
Burundi	1·52	2·72	3·49	4·56	2·65	4·57	7·95	4·62	6·18	5·38	8·00
Cambodia	8·56	9·48	8·11	9·08	8·01	8·47	8·69	7·81	13·67	6·85	8·63
Cameroon	1·73	2·79	2·32	2·26	4·13	2·03	3·17	1·94	1·54	2·46	4·70
Central African Republic	2·63	3·31	4·26	4·77	2·46	7·92	2·56	6·47	4·75	2·95	3·49
Chad	2·33	2·31	3·54	2·28	1·22	1·86	2·17	3·22	3·69	1·26	2·70
China (People's Republic of)	0·04	0·06	0·07	0·09	0·12	0·12	0·15	0·06	0·05	0·03	0·03
Comoros	1·79	2·44	0·33	1·16	1·26	1·21	0·60	1·47	3·06	2·65	2·18
Congo	0·46	2·00	3·15	5·16	3·97	5·17	3·30	5·88	4·87	2·27	3·41
Côte d'Ivoire	4·31	3·72	3·94	4·14	5·47	8·70	6·99	10·31	7·96	8·41	8·82
Democratic People's Republic of Korea	0·00	0·00	0·00	0·00	0·00	0·00	0·00	0·05	0·00	0·08	0·02
Democratic Republic of the Congo	1·46	1·13	2·15	1·70	1·91	3·08	3·18	5·26	3·71	3·41	2·75
Djibouti	2·76	1·67	12·97	13·17	15·71	13·90	6·55	7·08	8·29	3·38	11·17
Egypt	0·87	1·68	1·19	1·20	1·28	1·28	0·63	0·49	0·12	0·08	0·06
Equatorial Guinea	0·49	0·42	8·28	4·82	1·34	8·00	2·48	4·58	1·83	0·52	0·16
Eritrea	3·69	7·59	8·85	4·52	5·84	7·04	6·40	7·15	2·73	5·91	3·84
Ethiopia	2·49	3·21	4·15	4·25	7·60	7·65	5·63	11·36	10·10	6·96	8·48
Gabon	1·71	3·64	1·96	4·02	5·93	4·38	7·82	4·51	4·32	3·67	4·62
Gambia	5·12	12·07	15·63	6·68	4·85	4·70	9·13	8·83	8·55	6·71	12·21
Ghana	2·18	3·80	3·36	3·37	4·22	4·08	6·05	4·07	6·14	4·74	7·56
Guatemala	1·54	1·31	1·18	0·97	1·84	3·71	3·30	2·78	3·70	2·77	3·62

TRIAL MODE - a valid license will remove this message. See the keywords property of this PDF for more information.

Guinea	3·69	4·42	3·98	3·64	3·09	4·20	3·41	3·57	4·42	3·16	4·62
Guinea-Bissau	0·21	0·93	4·56	5·56	6·18	4·39	8·28	9·26	7·95	2·51	1·12
Haiti	9·55	11·55	18·87	28·67	30·42	20·09	27·73	29·37	28·47	26·99	27·18
India	0·21	0·22	0·19	0·17	0·29	0·19	0·36	0·35	0·48	0·32	0·38
Indonesia	0·44	0·56	0·54	0·39	0·20	0·20	0·35	0·27	0·34	0·37	0·41
Iraq	0·02	0·02	0·00	0·00	0·01	0·03	0·04	0·00	0·03	0·15	0·15
Kenya	6·53	9·41	5·59	8·23	11·75	13·70	18·37	23·33	26·10	31·37	33·39
Kyrgyzstan	0·38	1·03	1·38	0·64	1·32	2·14	0·97	0·81	1·30	0·98	1·24
Lao People's Democratic Republic	0·81	1·11	1·67	0·91	1·45	1·38	2·17	2·13	3·26	1·78	2·60
Lesotho	8·39	9·02	9·61	10·74	21·83	31·47	35·15	52·65	67·62	75·73	60·08
Liberia	0·86	1·54	3·81	2·67	5·91	8·91	9·24	14·98	15·23	11·62	15·90
Madagascar	2·68	2·74	1·12	1·58	1·39	1·55	1·48	2·68	1·42	2·80	2·71
Malawi	10·39	17·73	13·46	13·79	19·08	15·84	13·81	14·06	21·33	26·20	23·61
Mali	1·43	2·72	3·39	4·16	5·89	7·23	6·30	6·35	5·89	6·92	6·39
Mauritania	1·02	3·49	2·51	4·77	4·10	4·41	0·58	0·12	1·47	1·78	1·95
Mexico	0·06	0·08	0·02	0·01	0·03	0·02	0·02	0·05	0·08	0·10	0·08
Morocco	0·38	0·27	0·37	0·33	0·47	0·26	0·34	0·45	0·36	0·20	0·16
Mozambique	6·25	12·05	10·25	15·55	18·85	19·32	18·70	24·21	23·21	22·17	23·32
Myanmar	0·26	0·29	0·48	0·20	0·27	0·30	0·29	0·68	0·58	0·71	1·29
Nepal	2·94	3·04	2·98	3·75	1·76	1·16	1·80	1·76	1·98	2·46	3·67
Niger	0·63	1·33	1·63	2·02	1·56	2·43	3·27	1·32	2·67	1·53	2·62
Nigeria	1·25	1·65	2·13	3·10	4·07	4·77	5·44	5·96	5·47	6·12	8·01
Pakistan	0·42	0·15	0·12	0·18	1·76	0·11	0·51	0·52	0·52	0·60	1·06
Papua New Guinea	4·46	6·57	7·15	13·59	17·17	19·03	15·19	12·53	14·11	18·60	18·52
Peru	0·34	0·32	0·61	0·68	0·76	1·01	1·44	1·31	0·93	0·77	0·48
Philippines	1·18	1·12	2·10	1·57	1·03	0·88	1·11	0·83	1·10	0·88	0·87
Rwanda	5·66	10·69	12·11	18·49	23·93	25·33	24·42	42·34	41·33	39·92	30·70
Sao Tome and Principe	0·00	0·31	1·61	3·25	5·31	5·85	1·75	2·92	12·50	5·81	12·96
Senegal	3·50	5·75	4·68	4·58	4·10	4·18	5·69	6·60	7·59	4·15	8·30
Sierra Leone	2·29	2·33	5·20	3·22	4·27	5·08	6·42	8·80	7·29	9·68	8·09
Solomon Islands	1·86	0·00	2·55	0·02	3·20	0·00	1·32	0·65	2·47	1·33	1·81
Somalia	0·39	0·30	1·84	1·93	2·47	1·19	0·74	3·01	2·73	1·47	2·54
South Africa	3·01	4·52	5·87	6·99	12·48	14·54	20·37	24·21	24·22	24·56	22·11

TRIAL MODE - a valid license will remove this message. See the keywords property of this PDF for more information.

South Sudan									7·19	8·75	8·78
Sudan	0·10	0·21	1·28	1·92	0·94	1·40	1·11	3·04	1·28	0·78	0·97
Swaziland	20·14	10·70	51·93	28·37	38·47	35·86	47·29	111·95	109·10	99·07	107·14
Tajikistan	0·59	0·93	0·62	0·33	1·28	1·12	0·75	2·33	1·00	2·22	1·54
Tanzania	4·13	6·56	8·16	8·85	11·99	13·78	14·13	19·59	18·37	19·51	21·86
Togo	2·35	2·48	2·74	3·12	3·94	2·05	5·69	3·69	3·17	2·02	4·07
Turkmenistan	0·21	0·07	0·14	0·10	0·19	0·33	0·22	0·09	0·18	0·16	0·12
Uganda	5·93	14·75	10·57	12·24	15·57	12·60	17·36	21·85	22·64	27·57	25·13
Uzbekistan	0·88	0·22	0·34	0·25	0·30	0·13	0·27	0·13	0·26	0·63	0·14
Viet Nam	0·98	0·65	0·51	0·73	1·05	0·87	0·93	0·97	1·29	0·92	0·88
Yemen	0·46	0·65	1·11	0·87	0·72	0·51	1·30	1·40	0·57	0·72	1·84
Zambia	13·14	22·49	25·87	22·31	26·67	35·50	33·14	36·38	45·52	43·45	51·49
Zimbabwe	7·21	7·46	11·06	7·56	18·38	8·57	13·67	22·48	17·63	34·07	27·03

TRIAL MODE - a valid license will remove this message. See the keywords property of this PDF for more information.

Appendix Table 10: Disbursements for reproductive, maternal, newborn and child health to 75 priority recipient countries by area of spending, 2003–13, constant 2013 USD millions

	2003		2004		2005		2006		2007		2008		2009		2010		2011		2012		2013	
**General budget support**	**149**	**3%**	**118**	**3%**	**114**	**2%**	**132**	**2%**	**128**	**2%**	**162**	**2%**	**235**	**2%**	**181**	**2%**	**138**	**1%**	**149**	**1%**	**78**	**1%**
**Sector budget support**	**7**	**0%**	**12**	**0%**	**26**	**0%**	**32**	**1%**	**145**	**2%**	**120**	**1%**	**66**	**1%**	**81**	**1%**	**58**	**0%**	**45**	**0%**	**13**	**0%**
**Basket-funding**	**23**	**1%**	**70**	**2%**	**51**	**1%**	**57**	**1%**	**60**	**1%**	**180**	**2%**	**136**	**1%**	**320**	**3%**	**209**	**2%**	**48**	**0%**	**2**	**0%**
**Projects**	**4128**	**96%**	**4122**	**95%**	**5878**	**97%**	**6236**	**97%**	**7529**	**96%**	**7988**	**95%**	**10140**	**96%**	**10716**	**95%**	**11536**	**97%**	**12347**	**98%**	**13896**	**99%**
Malaria (specific to MNCH)	11	0%	24	1%	77	1%	75	1%	101	1%	101	1%	165	2%	117	1%	66	1%	44	0%	107	1%
Malaria (not specific to MNCH)	76	2%	115	3%	215	4%	308	5%	292	4%	509	6%	899	9%	1016	9%	852	7%	1165	9%	1172	8%
Immunisation	448	10%	463	11%	551	9%	690	11%	1266	16%	1057	13%	1194	11%	1392	12%	1625	14%	1857	15%	2556	18%
Other child health activities	206	5%	206	5%	323	5%	304	5%	330	4%	312	4%	374	4%	412	4%	312	3%	297	2%	399	3%
Nutrition	264	6%	266	6%	358	6%	340	5%	190	2%	250	3%	583	6%	571	5%	505	4%	705	6%	882	6%
HIV (specific to RMNCH)	7	0%	16	0%	86	1%	56	1%	127	2%	60	1%	30	0%	61	1%	39	0%	71	1%	73	1%
HIV (not specific to RMNCH)	438	10%	681	16%	1673	28%	2084	32%	2638	34%	2814	33%	2979	28%	3435	30%	3853	32%	3687	29%	3752	27%
Reproductive health	624	14%	536	12%	718	12%	697	11%	936	12%	1096	13%	1470	14%	1424	13%	1837	15%	1927	15%	2106	15%
Family planning	402	9%	137	3%	360	6%	304	5%	299	4%	452	5%	633	6%	599	5%	745	6%	748	6%	886	6%
Sexually transmitted infections	693	16%	732	17%	116	2%	48	1%	61	1%	20	0%	111	1%	132	1%	95	1%	65	1%	83	1%
Sexual health	2	0%	5	0%	1	0%	5	0%	5	0%	14	0%	63	1%	58	1%	43	0%	45	0%	49	0%
General health care/health systems	958	22%	942	22%	1399	23%	1324	21%	1284	16%	1303	15%	1638	15%	1498	13%	1566	13%	1736	14%	1832	13%
**Total**	**4307**		**4322**		**6069**		**6457**		**7862**		**8450**		**10577**		**11299**		**11940**		**12588**		**13989**	

Appendix figure 1: Targeting of ODA+ for CH per child to under-5 mortality rates

Excludes recipient countries with population <250,000 as these had disbursements that were very high per person but low in absolute terms.

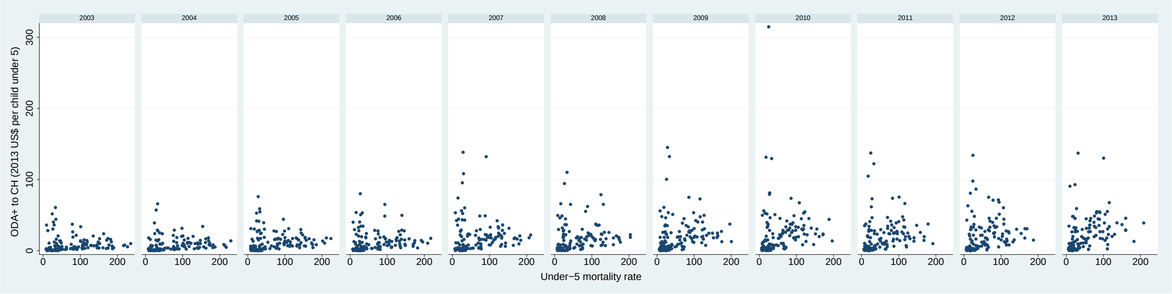

Appendix figure 2: Targeting of ODA+ for MNH per live birth to maternal mortality ratios

Excludes recipient countries with population <250,000 as these had disbursements that were very high per person but low in absolute terms.

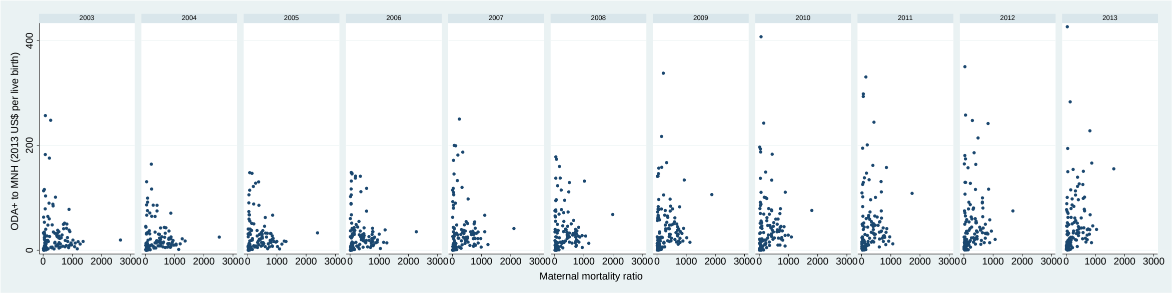

Appendix figure 3: Targeting of ODA+ for R* per woman aged 15–49 to HIV prevalence

Excludes recipient countries with population <250,000 as these had disbursements that were very high per person but low in absolute terms.

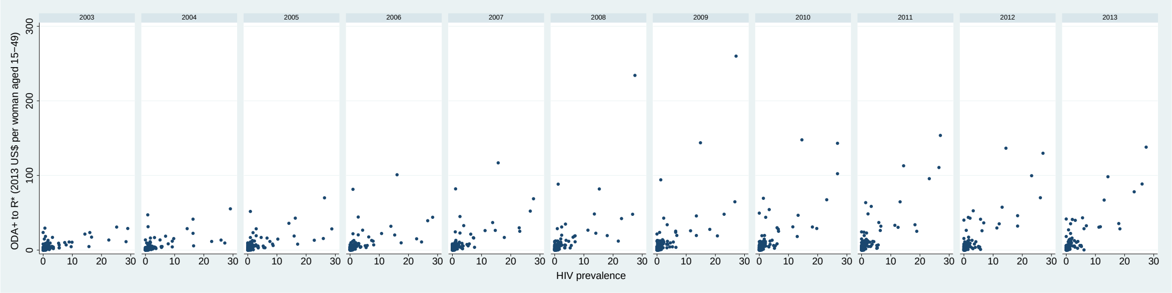

Appendix figure 4: Targeting of ODA+ for R* per woman aged 15–49 to female life expectancy at birth

Excludes recipient countries with population <250,000 as these had disbursements that were very high per person but low in absolute terms.

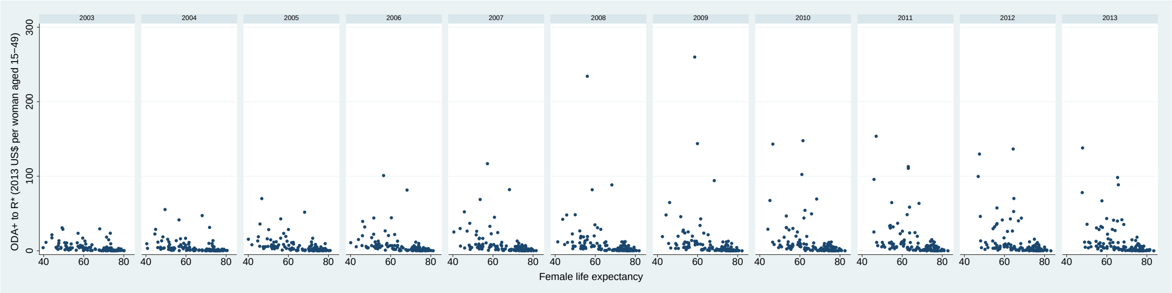

TRIAL MODE - a valid license will remove this message. See the keywords property of this PDF for more information.
